# Households as hotspots of Lassa fever? Assessing the spatial distribution of Lassa virus-infected rodents in rural villages of Guinea

**DOI:** 10.1080/22221751.2020.1766381

**Published:** 2020-05-27

**Authors:** Joachim Mariën, Giovanni Lo Iacono, Toni Rieger, Nfaly Magassouba, Stephan Günther, Elisabeth Fichet-Calvet

**Affiliations:** a Department of Clinical Sciences/Outbreak Research Team, Institute of Tropical Medicine, Antwerp, Belgium; b School of Veterinary Medicine, University of Surrey, Guildford, UK; c Bernhard-Nocht-Institute for Tropical Medicine, Hamburg, Germany; d Laboratoire des Fièvres Hémorragiques, Nongo, Conakry, Guinea

**Keywords:** Lassa virus, *Mastomys natalensis*, spatial distribution, cluster analyses, phylogeny, Guinea, West Africa

## Abstract

The Natal multimammate mouse (*Mastomys natalensis*) is the reservoir host of Lassa virus (LASV), an arenavirus that causes Lassa haemorrhagic fever in humans in West Africa. While previous studies suggest that spillover risk is focal within rural villages due to the spatial behaviour of the rodents, the level of clustering was never specifically assessed. Nevertheless, detailed information on the spatial distribution of infected rodents would be highly valuable to optimize LASV-control campaigns, which are limited to rodent control or interrupting human–rodent contact considering that a human vaccine is not available. Here, we analysed data from a four-year field experiment to investigate whether LASV-infected rodents cluster in households in six rural villages in Guinea. Our analyses were based on the infection status (antibody or PCR) and geolocation of rodents (*n *=* *864), and complemented with a phylogenetic analysis of LASV sequences (*n *=* *119). We observed that the majority of infected rodents were trapped in a few houses (20%) and most houses were rodent-free at a specific point in time (60%). We also found that LASV strains circulating in a specific village were polyphyletic with respect to neighbouring villages, although most strains grouped together at the sub-village level and persisted over time. In conclusion, our results suggest that: (i) LASV spillover risk is heterogeneously distributed within villages in Guinea; (ii) viral elimination in one particular village is unlikely if rodents are not controlled in neighbouring villages. Such spatial information should be incorporated into eco-epidemiological models that assess the cost-efficiency of LASV control strategies.

## Introduction

Household characteristics can affect the distribution of zoonotic pathogens when they influence the habitat suitability of the animal hosts [1,2]. When these characteristics (e.g. food storage, waste disposal, building materials, presence of pets) are unevenly distributed in the environment, focal clusters or hotspots of disease transmission can occur, defined as areas of elevated transmission efficiency and spillover risk [3]. Examples of zoonotic diseases that cluster in the human environment are leptospirosis, rabies, West Nile fever and bubonic plague [4–6]. Hotspot detection is important to understand the drivers of disease transmission and has successfully increased the cost-effectiveness of control programs [7,8]. Failure to recognize and correctly implement this spatial heterogeneity into eco-epidemiological models may result in poor predictions of disease outbreak probabilities, as well as missed opportunities to optimize control strategies [9,10].

Hotspots are also suggested to exist for Lassa virus (LASV), an arenavirus that causes Lassa haemorrhagic fever (LF) in humans [11–13]. The disease is limited to West Africa where most cases are found in poor, rural villages. Although LF is likely underreported in this area, an often-cited report concluded that between 200,000 and 300,000 infections occur each year with a fatality rate of 1–2% [14]. For a long time, the Natal multimammate mouse (*Mastomys natalensis*) was assumed to be the sole reservoir host of the virus, but recent field studies suggest that other rodents may also serve as reservoirs (e.g. LASV RNA was found in *Mastomys erythroleucus* and *Hylomyscus pamfi* in Nigeria) [15,16]. Nevertheless, as *M. natalensis* is the main species trapped inside houses of rural villages (the rodent typically comprises more than 90% of all species trapped indoor), we can assume that humans mainly become infected after contact with this species [17,18]. Spillover can happen through ingestion of contaminated food or water, inhalation of aerosolized viral particles, or direct consumption of the rodent [19–21]. Human-to-human transmission is thought to be uncommon, although certain activities may increase the likelihood of transmission, such as nosocomial exposure or close contact in the same household, likely arising from super-spreading events with potentially significant impact [22,23].

LASV-infected rodents are suggested to cluster within rural villages due to the heterogeneous availability of food between houses and the non-territorial behaviour of *M. natalensis* [11,12]. In West Africa, these rodents live communally in burrows under the floors and walls of houses or outside in patches of cultivated land, where they search for food and shelter [17,24]. Their density increases significantly inside houses during the dry season when fields are left fallow and the rodents are attracted by crops that are stored inside [25]. Home ranges are relatively small (∼650 m^2^) but can completely overlap when densities increase due to the animal’s promiscuous mating behaviour and lack of territoriality [26,27]. Consequently, contact rates between *M. natalensis* are density-dependent and virus transmission is likely to increase at foci where densities are higher [28]. We therefore expect that variations in household characteristics (mainly driven by differences in food availability) result in focal areas with high rodent density, LASV prevalence and spillover risk to humans [29].

Detailed information on the spatial distribution of LASV-infected rodents would be highly valuable to optimize LASV-control strategies, which are currently (in the absence of a human vaccine or effective drug) limited to rodent control and human behavioural changes [30]. The main objective of this study was to establish whether LASV-infected rodents cluster in households in rural villages. We tested three hypotheses: (i) LASV infected *M. natalensis* (viral RNA and/or antibody positive) are unevenly distributed throughout the rodent population in the villages, (ii) *M. natalensis* are unevenly distributed throughout houses in the villages, (iii) LASV-sequences of animals captured in the same house are genetically more similar than sequences of animals captured in different houses of the same village. In addition, we investigated if LASV-sequences of infected rodents group together in time and space within and between villages.

## Methods

### Study sites and experimental setup

**Figure 1.**
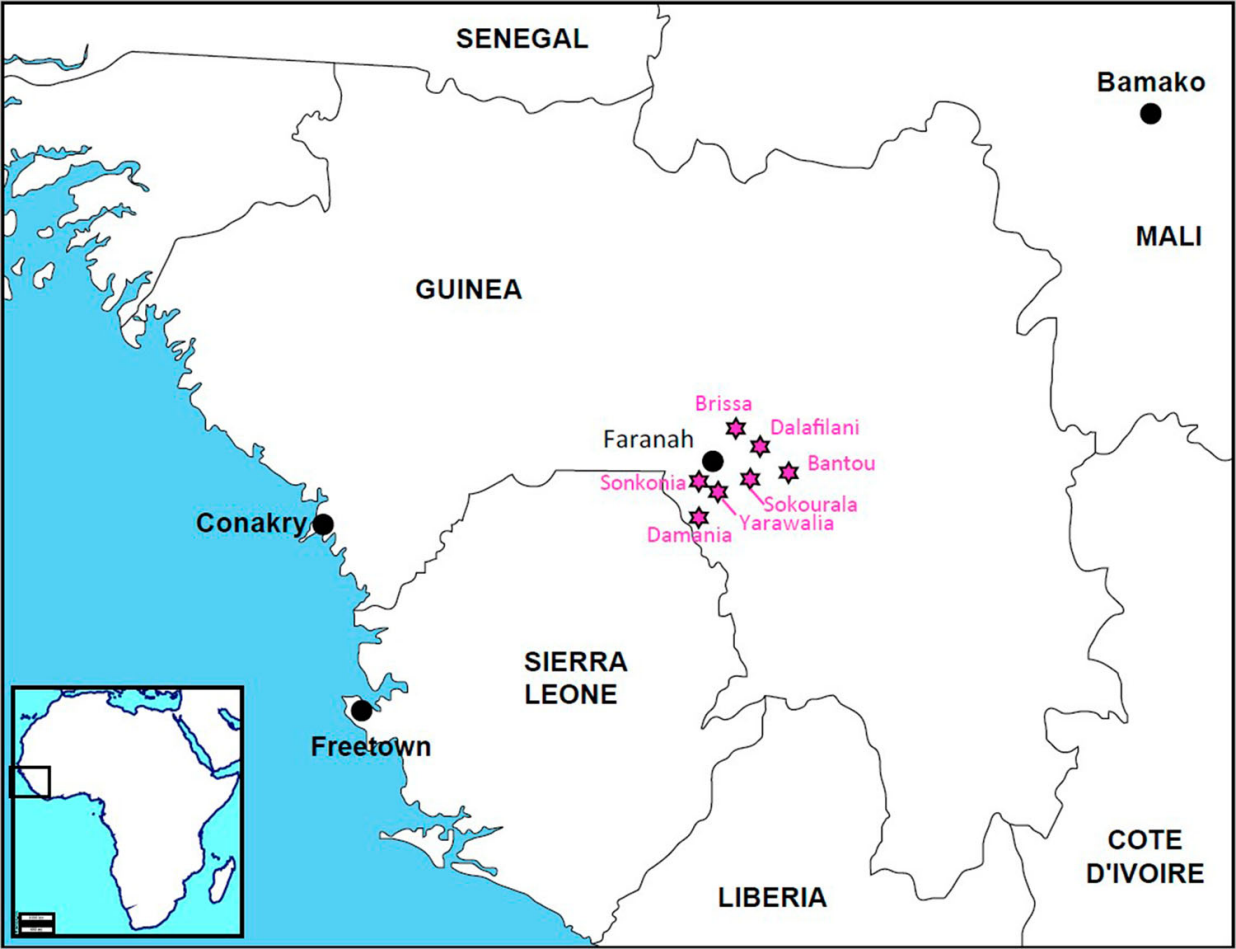
Map of Guinea showing the location of Faranah district and the six villages included for the cluster analyses [Brissa (10°13.010′ N; 10°41.326′ W), Dalafilani (10°08.590′ N; 10°36.303′ W), Damania (09°48.410′ N; 10°51.796′ W), Sokourala (10°03.407′ N; 10°39.950′ W) and Sonkonia (09°54.763′ N; 10°47.888′ W)], and Bantou from which the LASV strain was used as reference for the IFA slides and the phylogenetic analysis.

We used data from a four-year rodent-control experiment performed in the prefecture of Faranah (Upper Guinea). In short, six rural villages were randomly grouped into treatment (Brissa, Dalafilani and Yarawalia) and control (Damania, Sokourala and Sonkonia) villages (Figure 1). All villages met to the following criteria: *M. natalensis* was abundantly present in the houses (>95% of captures is *M. natalensis*), high LASV seroprevalence in the rodent population (>20%), short travel distance from Faranah (<45 min drive) and small population size (<1000 inhabitants). Rodenticides were distributed once a year (for 10–30 days between November 2013 and March 2017) in all treatment villages. The interventions were carried out during the dry season (November–April) when rodents were assumed to aggregate in houses in search of food and shelter. Anticoagulant baits (Bromadiolone or Difenacoum) were distributed in baiting stations (Coral, 158 Ensystex Europe) and were placed in each house, resulting in 300–600 baiting stations per village. We refer to Sáez et al. [31] and Mariën et al. [30] for a detailed explanation of the experimental setup.

### Rodent trappings

Trapping sessions consisted of three consecutive trap nights, which were performed before and after intervention in the treatment villages and once a year in the control villages. For each trap night, Sherman live traps (Sherman Live Trap Co., Tallahassee, FL, USA) were placed in pairs in 60 rooms of 42–50 houses that were randomly chosen along a transect. The traps were baited (with a mixture of peanuts, dry fish and wheat flour) in the evening and checked the next morning. During the first three years, we only took coordinates from houses in which rodents were captured. In the last year, coordinates were taken from all houses in which traps were placed. Coordinates of houses (and rooms) were taken with a hand-held Garmin® GPS (accuracy 5 m). Trapped rodents were humanely killed (isoflurane) and necropsied in situ according to BSL3 procedures, as explained in [24,32]. Due to personnel safety issues, it was not possible to trap rodents in the villages Sokourala (years 2 and 3) and Sonkonia (year 2) during the Ebola epidemic in 2014–2015 [31].

### Viral RNA detection in blood and sequencing

During rodent autopsies, blood was collected and preserved under 2 biopsies: whole blood in Eppendorf tubes and dried blood on filter paper (SEROBUVARD – LABOCEA, France) in small re-sealable zipper bags with desiccant silica. Both were stored at −20°C. LASV screening was performed on all dried blood samples with two RT-PCRs used for Lassa diagnostic: one Lassa specific and one pan-arena test [33]. The testing was done on three pooled blood samples. Extractions were performed with the QIAmp RNA Mini Kit (Qiagen, Hilden, Germany). If positive, pools were split and retested individually with both RT-PCRs. To check whether dried blood on filter paper was a good carrier of viral RNA, whole blood from a fraction of the sampling was also tested using the same method. To perform a more reliable phylogeny than with short fragments issued from the diagnostic tests, we did additional PCRs on the glycoprotein (GPC) and nucleoprotein (NP) genes located on the S-segment. GPC was amplified with the forward primer LVS36+ (ACC GGG GAT CCT AGG CAT TT) and reverse primer OWS1000− (AGCATGTCACAGAAYTCYTCATCATG). NP was amplified using a nested RT–PCR protocol with the following primers: outer primers LVS 1607-fwd (GGTGTTGATGTTCTAAASACC) and LVS 2535-rev (GCCTGCATGTTGGATGGTGGC); nested primers LVS 1629-fwd (TGTCTCTGGGCAGCACTGCTC) and LVS 2429-rev (TGTTTGTCTCAGACACTCCYGGTG) [13]. All amplicons were confirmed by Sanger-sequencing in both directions. In total, we generated 95 partial GP and 84 partial NP sequences of LASV. Sequences were submitted to GenBank under accession numbers MT119462-MT119640. The sequences from *M. natalensis* used in our study are listed as supplementary file (“supplementary material: excel file sequences”).

### Phylogenetic analysis of LASV

Phylogeny was inferred by the Bayesian Markov Chain Monte Carlo method implemented in BEAST software [34]. To get a better estimation of the time of emergence with a longer fragment than partial fragment analysed separately, we merged the partial GP and NP in a combined phylogenetic analysis. In BEAUTI, the parameters are:

- Two partitions, GP 888 nt and NP 735 nt for 140 sequences. The substitution models, clock and trees are linked
- Eight taxa were defined according to the locality: Bantou, Brissa, Dalafilani, Damania, Madina Oula, Sokourala, Sonkonia, and Yarawalia.
- Tip dates at the nearest day
- Substitution model as GTR + gamma and codon partition with positions 1,2,3
- Strict (model 2a) or uncorrelated relaxed (model 2b) clock
- Coalescent tree with a constant size population
- MCMC = 50 M, echo states and log parameters every 50,000

The xml files issued from BEAUTI were run in BEAST, checked in TRACER and consensus trees were visualized through Fig Tree (BEAST packages, https://beast.community/programs).

### Serology

Vero cells infected with LASV strain Bantou 366 were spread on immunofluorescence slides, air dried, and acetone-fixed [35,36]. The Bantou strain was chosen because it is the closest one which has been isolated in BSL4. Whole blood samples were stored in tubes in −20°C and centrifuged. From each sample, 10 µl supernatant was diluted (1:20) in phosphate-buffered saline (PBS) and Triton 1%. If whole blood was missing, we eluted a blood spot on filter paper in PBS and 0.25% NH3. The diluted serum was incubated with the cells, and bound IgG was detected with anti-mouse IgG-fluoresceine isothiocyanate (Jackson ImmunoResearch). Signals were evaluated with a fluorescence microscope by two independent observers [35,36]. The serostatus was only confirmed if the two results matched, while uncertain samples were re-assessed on a new IFA-slide with infected and non-infected cells [36].

### Statistical analyses

#### Spatial clustering of LASV-RNA and antibody-positive animals in the rodent population

We first investigated if LASV-RNA and/or antibody-positive *M. natalensis* clustered within the rodent population of the villages. Because the GPS coordinates of empty (no rodents captured) houses were not noted during the first three years, we used a nearest-neighbour and spatial scan statistic, which are methods that examine local patterns of cases in the vicinity of other cases. The key advantage of these approaches is that they do not require the population of the rodents to be uniformly distributed, which is the case in our study as houses were not uniformly distributed in the villages [37,38]. We included antibody positive animals in our analyses as they can still be infectious (shedding in excretions or faeces) even if they tested negative for LASV-RNA in blood [39,40]. Furthermore, due to the short average lifespan of *M. natalensis* (three months) and the relatively long infectious period (min. 3 weeks in excretions and saliva), clustering of antibody-positive animals would suggest that infectious animals clustered at least a few weeks before sampling [41,42].

We first used the Cuzick-Edwards (nearest-neighbour) test to investigate the overall clustering of LASV-positive animals within the rodent population. Evidence of clustering would be obtained if we observed more cases among locations nearest each case than one would expect under the random labelling hypothesis [37]. Because different values of *q*-nearest neighbours (i.e. the number of neighbours included in the analysis) may generate different results, possibly indicating the scale of the clusters, we assessed *q* for two up to 10 nearest neighbours in steps of 2. Clusters with *q* > 10 were not considered because the number of sampled locations (number of rooms = 60) was relatively low. Furthermore, clusters were only assessed for trapping sessions where more than ten rodents were captured of which at least five tested positive for antibodies or PCR (otherwise the sample size was simply to low to calculate clusters of cases). We therefore had to discard all data from trapping sessions that were performed after anticoagulant rodenticide treatment. Monte Carlo permutations (*n* = 10,000) were run to infer if clustering of the field data was significantly higher (*p* ≤ .05) than in random simulations by using the “qnn-test” from the smacpod R-package [43].

After confirming that LASV-positive rodents were significantly clustered during several trapping sessions, we used the Kulldorff’s spatial scan statistic to characterize the local clusters (how many local clusters, where do they occur, how many rodents involved) [38]. This statistic searches for collection(s) of cases that are least consistent with the null hypothesis (i.e. the most likely clusters) through a variable scanning window over the area of interest. Significance values of clusters were obtained by running simulations with the “spscan-test” from the smacpod R-package [43].

#### Spatial clustering of rodents in houses

After screening for clusters of infected *M. natalensis* within the rodent populations, we investigated if some houses (rooms) contained more rodents than one would expect under the random labelling hypothesis. We again used Monte Carlo permutations to infer if numbers of rodents captured in individual rooms differed significantly from distributions obtained by random sampling (by reshuffling animals over all houses). Because coordinates of empty rooms (in which no *M. natalensis* were captured) were only taken during the last trapping year (2016–2017), we could only test the amount of clustering for the final year. Note that this analysis is possible because we placed exactly two traps in each room during three consecutive trap nights.

#### Genetic analyses

To test if LASV-sequences from the same village and year would group together, we performed a principal component analysis (PCA) with the genetic distance matrix (based on LASV sequences from infected rodents) as input variable. In this way, we could summarize the information from the high dimensional distance matrix (119 × 199) to calculate scores (the principal components) that summarize the information as efficiently as possible. The advantage of this approach is that it allows visualization of the genetic relationship between all LASV-sequences in two dimensions from which distinct groups can be observed.

Based on the LASV-genetic distance matrix, we also tested whether the genetic distance (*D_ij_*) is lower for pairs of PCR-positive rodents (*i* and *j*) captured in the same house versus pairs of PCR-positive rodents captured in different houses during the same trapping session. Because of dependency in the data (the genetic distance of each *D_ij_* pair is related to all other pairs that contain individuals *i* or *j*), we sorted the distances *D_ij_* to rodent *i* (fixed *i* and *j* changed) and calculated the average rank (*R_ij_*) of those rodents sharing a house with rodent *i*. We tested whether *R_ij_* is significantly lower than expected under a random distribution with the Wilcoxon signed-rank test.

We then used the LASV-genetic distance matrix to assess the correlation between the genetic and Euclidean distance of LASV-sequences that were obtained from rodents captured at the same trapping session. We calculated the Spearman rank correlation for each rodent *i* and used the Wilcoxon signed-rank test to assess if the obtained correlation coefficients differed significantly from zero.

## Results

### Spatial clustering of infectious rodents in the rodent population

From the 34 trapping sessions that were organized in villages around Faranah, 22 could be used to assess the spatial clustering of LASV-infected *M. natalensis* based on sample size considerations (Table 1). During these trapping sessions, a total of 864 *M. natalensis* were captured and tested on the presence of LASV-antibodies and viral RNA.

**Table 1.** Monte Carlo *p*-values (based on 1000 simulations) to infer if LASV infected *M. natalensis* (antibody and PCR positive) are significantly clustered in the rodent populations of the villages based on the Cuzick-Edwards test.

Village	Date	Positives^a^	Total^b^	*N*^c^ = 2	*N* = 4	*N* = 6	*N* = 8	*N* = 10
Brissa	14/12/2013	18	51	**<0**.**01**	**<0**.**01**	**<0**.**01**	**<0**.**01**	**<0**.**01**
Brissa	26/03/2015	9	25	**0**.**03**	0.20	0.07	0.19	NA^d^
Brissa	9/11/2015	22	56	0.10	0.35	0.46	0.31	0.10
Brissa	12/11/2016	13	35	0.44	0.07	0.11	0.07	0.27
Dalafilani	28/11/2013	10	39	0.25	**0**.**02**	0.25	0.15	0.13
Dalafilani	17/04/2014	5	11	0.88	0.55	NA	NA	NA
Dalafilani	15/01/2016	13	30	**<0**.**01**	**<0**.**01**	**0**.**02**	**<0**.**01**	**<0**.**01**
Dalafilani	16/01/2017	11	23	**0**.**02**	**0**.**01**	**0**.**01**	0.16	0.17
Damania	15/11/2013	30	50	0.64	0.63	0.53	0.36	0.15
Damania	25/03/2014	25	59	0.13	0.77	0.60	0.62	0.37
Damania	13/11/2015	17	28	0.12	**<0**.**01**	**<0**.**01**	**<0**.**01**	**<0**.**01**
Damania	16/11/2016	14	45	**<0**.**01**	**<0**.**01**	**<0**.**01**	**0**.**02**	**0**.**03**
Sokourala	3/04/2013	25	46	0.15	**<0**.**01**	**<0**.**01**	**<0**.**01**	**<0**.**01**
Sokourala	31/01/2017	24	46	**0**.**02**	**0**.**02**	**0**.**03**	0.09	0.29
Sonkonia	12/11/2013	13	46	0.10	**0**.**04**	0.09	0.26	0.52
Sonkonia	7/04/2014	11	58	0.52	0.35	0.68	0.62	0.42
Sonkonia	19/11/2015	12	53	0.41	0.33	0.76	0.64	0.62
Sonkonia	24/11/2016	18	41	0.85	0.55	0.67	0.65	0.65
Yarawalia	20/11/2013	16	44	**<0**.**01**	0.06	0.07	**0**.**02**	0.27
Yarawalia	13/04/2014	7	17	0.15	0.05	**<0**.**01**	NA	NA
Yarawalia	21/03/2015	8	36	0.23	0.21	0.27	0.38	NA
Yarawalia	20/11/2016	14	25	**0**.**05**	**<0**.**01**	**<0**.**01**	**<0**.**01**	**<0**.**01**
All		335	864					

^a^Total number of LASV antibody or PCR positive *M. natalensis*.

^b^Total number of *M. natalensis* captured per trapping session in the villages.

^c^Number of nearest neighbours (*q*).

^d^Not applicable; bold numbers were considered to be significant.

Significant overall clustering of LASV infected *M. natalensis* (antibody or RNA-positive) was observed in 13 of the 22 trapping sessions, as Monte Carlo *p*-values were lower than .05 for at least one *q* nearest neighbour based on the Cuzick-Edwards test (Table 1). From these 13 trapping sessions, four clustered significantly for all *q* nearest neighbours. Clusters were also observed when we assessed the antibody (supplementary data: S table 1) or viral RNA (S Table 2) results separately.

**Figure 2.**
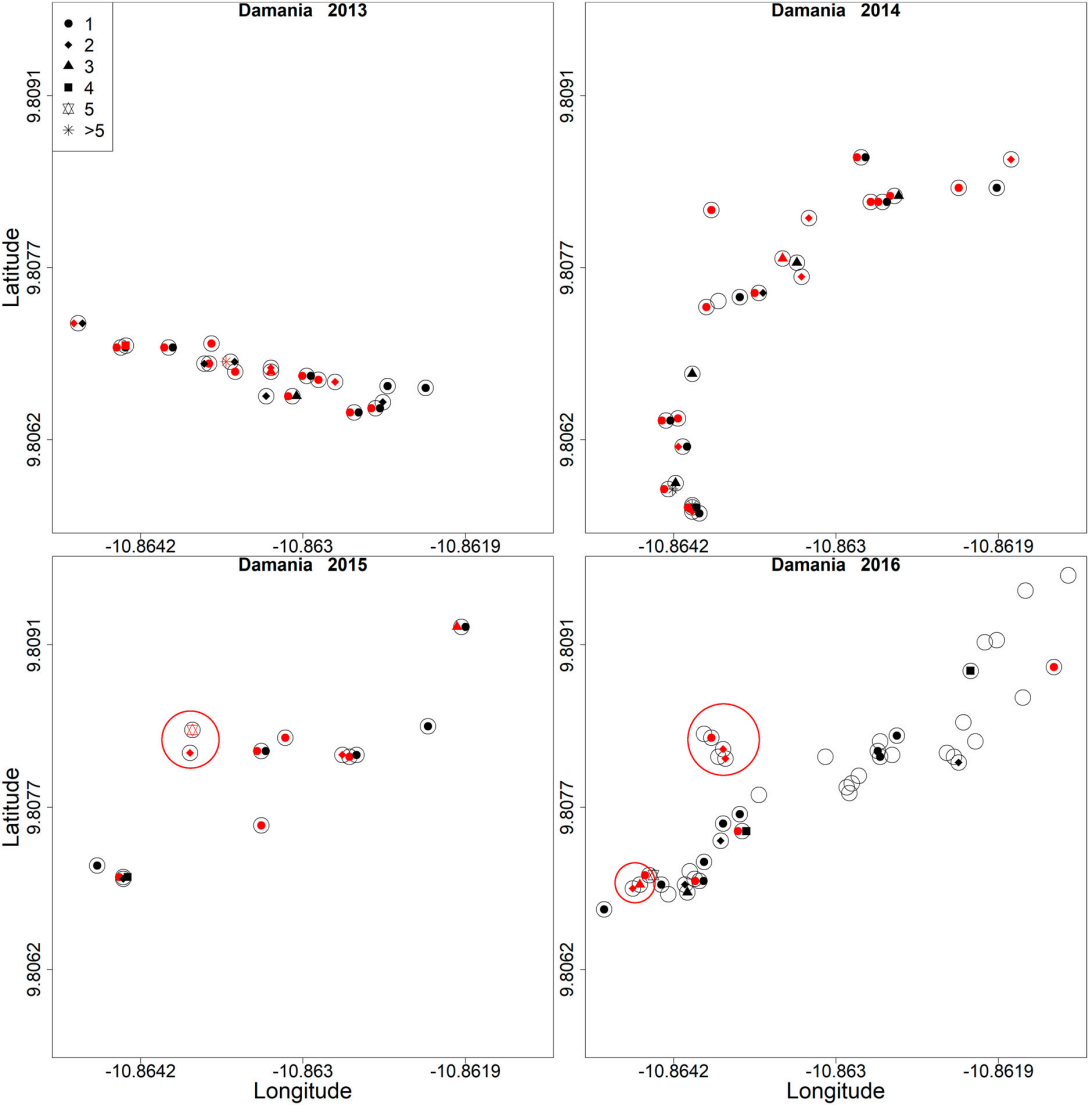
Position of houses (rooms) in the village of Damania where traps were placed. Empty circles represent houses (rooms) where no *M. natalensis* were captured and circles with dots represent houses where *M. natalensis* were captured. Red dots represent LASV-infected individuals (antibody or PCR-positive) and black dots uninfected ones. Red circles represent significant clusters of cases based on the spatial statistic scan. Coordinates of houses without rodents were only taken in 2016.

Significant local clusters of LASV infected *M. natalensis* (antibody or RNA-positive) were observed during ten trapping sessions based on the spatial scan statistic (Table 2; see supplementary tables 2 and 3 for antibody and PCR results only). We found one or two significant clusters per trapping session. Figure 2 represents the geolocations and infection statuses of rodents in Damania and the local clusters; similar figures can be found for the other villages and for antibody and PCR results separately in the supplementary material (Figure 2 and S Fig 1–17)

**Table 2.** List with significant local clusters based on the Kulldorff’s spatial scan statistic for *M. natalensis* that were infected (antibody or PCR-positive) with LASV.

Village	Date	*P*-values	Pos/neg^a^
Brissa^b^	14/12/2013	.05	5/0
Brissa^c^	14/12/2013	.05	7/2
Dalafilani	15/01/2016	.03	5/0
Damania	13/11/2015	.04	7/0
Damania^b^	16/11/2016	.03	5/0
Damania^c^	16/11/2016	.03	5/0
Sokourala	3/04/2013	.01	10/0
Sokourala	31/01/2017	.03	6/1
Yarawalia	13/04/2014	.02	5/1
Yarawalia	20/11/2016	<.01	10/0

^a^Number of positive versus negative animals that are located within the cluster (e.g. the first cluster in Brissa contained 5 positive and zero negative animals).

^b,c^Indicates that two clusters were found during the same trapping session.

### Spatial clustering of rodents in houses

**Figure 3.**
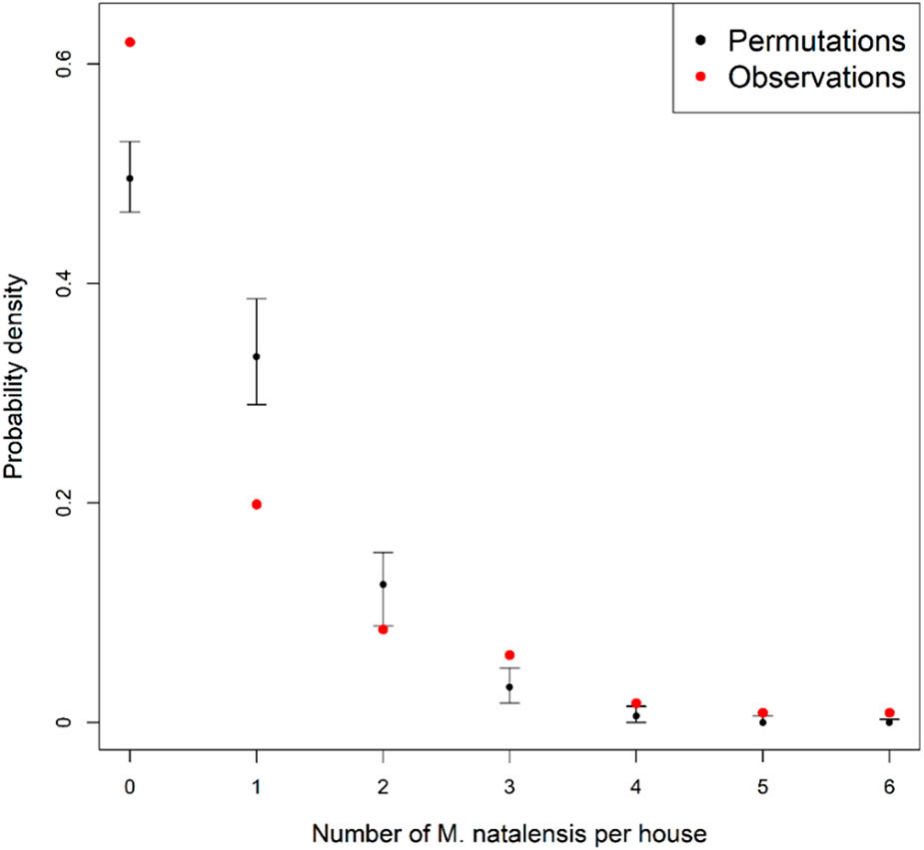
Probability that 0–6 *M. natalensis* were captured in a house (room) based on field observations (red) and random permutations (black) in rural villages in Guinea. Black bars represent 95% confidence intervals of probability density estimations.

*M. natalensis* clustered significantly in the houses, as the probability of trapping zero or more than two animals in the same room over three consecutive trapping sessions was significantly higher than expected by random sampling (Monte Carlo *p*-values <.0001) (Figure 3).

### Genetic analyses of LASV strains

The phylogenetic analysis suggests a dichotomy in LASV emergence between four villages where the virus first appeared between 1945 and 1968 (95% confidence intervals), and two villages where the virus appeared between 1977 and 1992 (95% confidence intervals) (S fig 18). The village of Sonkonia in particular shows a very disjointed emergence of the virus from Yarawalia and Damania, which are located only 10 and 15 km away respectively.

**Figure 4.**
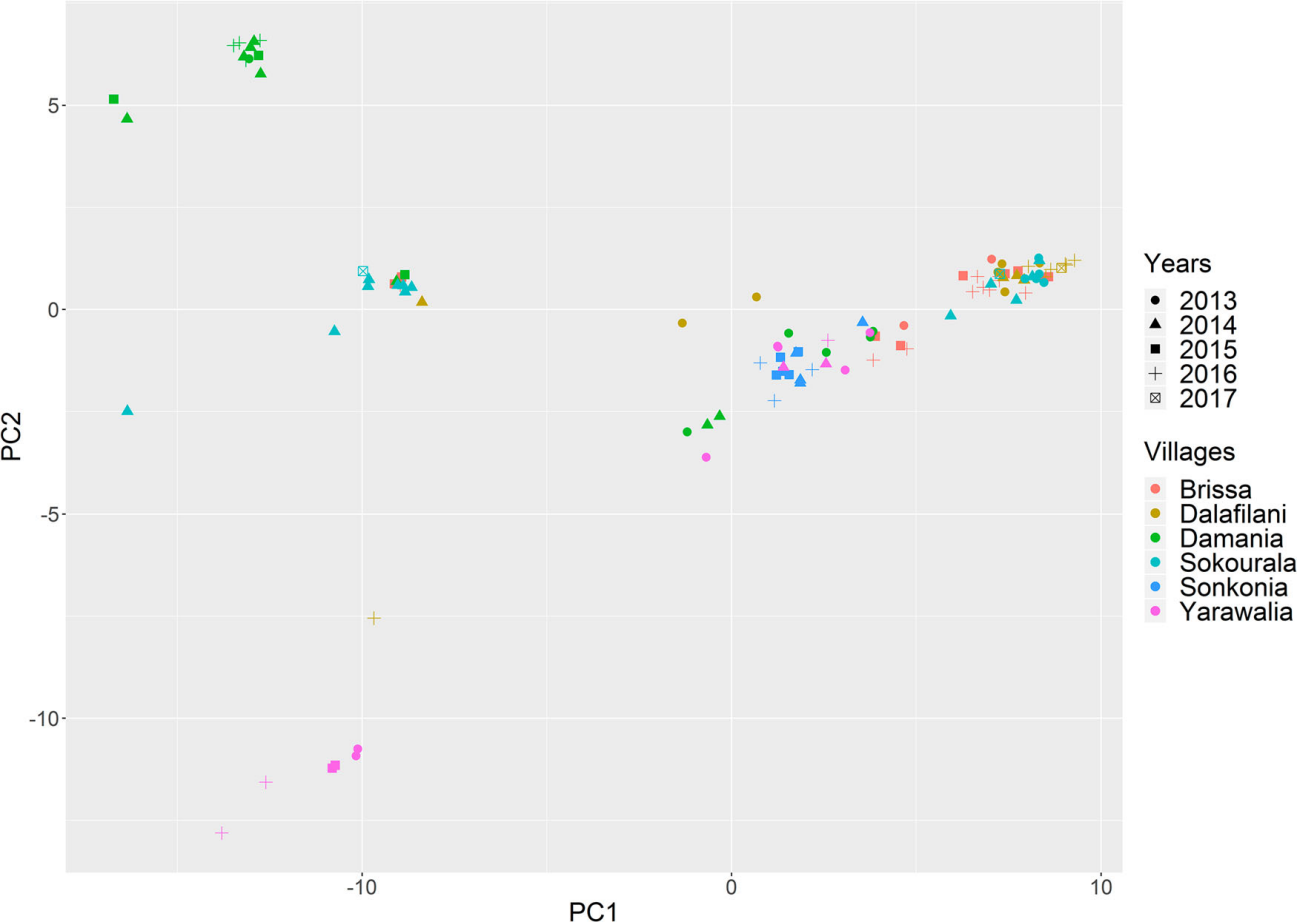
Principal component analysis on the genetic distance matrix of LASV sequences derived from captured *M. natalensis* in Guinea. *X*-axis represents principal component 1 and *Y*-axis represents principal component 2. Different symbols represent different years and colours different villages.

The phylogenetic tree and PCA analyses show that sequences in a specific village are polyphyletic, although there is overall subgrouping of strains at the village level (S fig 19). Only two villages (Yarawalia and Damania) contained a distinct group of strains that were not found elsewhere. Sequences of animals that were trapped in the same village but at different years were often grouped together; this was the case for both rodent elimination and control villages (Figure 4).

**Figure 5.**
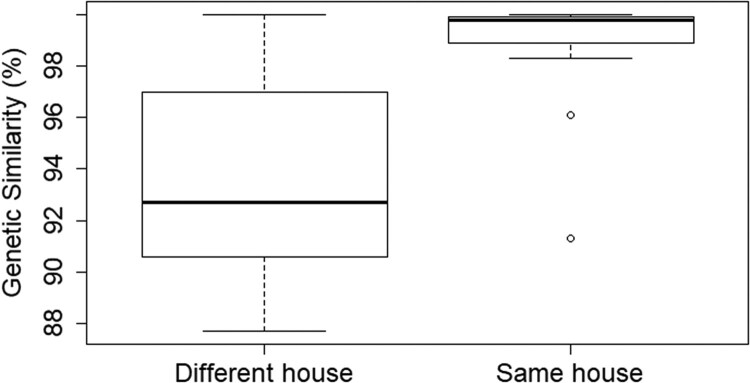
Boxplots representing the difference in mean genetic distance of LASV-sequences (100% = completely similar) obtained from *M. natalensis* captured in the same house or in different houses from the same village and year.

LASV sequences of animals that were captured in the same house were significantly more related than sequences of animals that were captured in different houses during the same trapping session (Wilcoxon test, median_same_house_ = 99.8, median_different_house_ = 92.7, *p* < .0001) (Figure 5). Correspondingly, a weak but significant negative correlation was found between the genetic and Euclidean distance for LASV-sequences of animals that were captured during the same trapping session (Wilcoxon test, median correlation coefficient = −0.47, *p* = .0029) (S fig 20).

## Discussion

Our study suggests that *M. natalensis* currently or previously infected with LASV tend to cluster in a few households in rural villages in Guinea. Although we did not investigate the household characteristics that may cause these hotspots, it was clear that the majority of infected animals were trapped in a few houses (∼20%) and most houses (∼60%) were rodent-free at a specific point in time. Our genetic data also shows that LASV-infectious rodents are more likely to infect rodents from the same (or neighbouring) house than animals from distant houses, suggesting the non-homogeneous mixing of animals at the village level.

Spatial clustering of LASV-infected rodents is consistent with predictions from previous studies and what is known about the ecology of virus and host [11,12]. When houses differ in the amount of food that is stored, or more generally in overall domestic organization (e.g. internal hygiene or rodent proofing), one can expect that some houses are more likely to attract *M. natalensis* than others [44]. This variation together with the observation that *M. natalensis* is a non-territorial social animal can explain why capture rates of *M. natalensis* are not randomly distributed over houses in the villages, as animals might actively search for food or conspecifics (e.g. to groom or mate) in already occupied houses [27,45]. Focal hotspots of infected *M. natalensis* can arise because arenavirus transmission is mainly direct and increases with rodent density [28,30,36,46]. An interesting observation is that consistent clustering across all nearest neighbours (4/22 trapping session, Table 1) always occurred during the beginning of the dry season (November–January). During this period, *M. natalensis* is assumed to stay in the houses for food and shelter, as the crops are stored inside and the fields are bare [47,48]. We also found that antibody-positive (but LASV RNA-negative) animals clustered significantly during several trapping sessions (S table 2). Antibodies against arenaviruses usually remain lifelong after infection in *M. natalensis* and the absence of viral RNA in blood suggests that these animals became infected more than two weeks before they were captured [40,41]. Clustering of antibody-positive animals suggests that home ranges of *M. natalensis* remain constant for several weeks, which corresponds to observations of capture-mark-recapture studies in Tanzania [25,45,49].

One limitation of our study is that we cannot directly link clusters of infected rodents to LASV-infection risk in humans. Although several attempts have been made in other studies, a significant link between housing characteristics, rodent density and LASV spillover risk has not yet been found [44]. Nevertheless, given that human LF cases peak when *M. natalensis* enters the houses in the dry season, we expect that such a causal link exists. Our results therefore suggest that control efforts aiming to rapidly reduce LASV spillover risk could focus on clusters of houses with (infected) rodents. To detect such hotspots on sight, one obvious indicator would be “the presence of rodent burrows” which was directly associated with past cases of human LF in Sierra Leone [50]. Correspondingly, camera trap images showed that *M. natalensis* can be very abundant around burrows in houses in Guinea [51]. Targeting houses with a lower infection risk (e.g. with no obvious signs of rodent burrows or faeces) can be done by using preventive methods that keep rodents away, such as storing food in airtight containers or repairing holes in the walls or roof [25,31]. Our results also indicate that spatial heterogeneity should be incorporated into eco-epidemiological models that aim to predict the effect of different control strategies at the village level [30].

Similar to the results of Fichet-Calvet et al. (2016) [13], we found that LASV strains circulating in a specific location are diverse and polyphyletic with respect to neighbouring villages. Nevertheless, we also observed that most strains group together at the sub-village level (e.g. in households or neighbouring houses) and persist over time in the same village. In addition, we found two clusters of strains that occur in one village only. These patterns suggest that LASV evolution in the natural reservoir is characterized by a combination of stationary circulation within a village and virus movement between villages. The latter process is exemplified by the more recent emergence of LASV in Sonkonia (S Fig 18), which we assume to be founded later in comparison to the other villages. Another interesting observation was that most strains could persist in villages where we performed rodent elimination, while we expected that a drop in population density would increase the extinction probability of these strains (genetic bottleneck effect). This result can be explained by the survival of chronically infected animals that ensure viral persistence during the critical host density period [46].

The latter results are relevant for rodent control strategies aiming to permanently eliminate LASV in the villages (in contrast to a fast but temporary reduction in spillover risk only). First, they imply that viral elimination by rodent control is difficult as several strains may persist during low host densities, so a sufficiently high percentage of rodents must be eliminated before LASV extinction may occur. Second, they imply that reinvasion from neighbouring villages is likely when extinction would be achieved. We therefore recommend that rodent control programs (aiming to eliminate LASV permanently in a certain area) should be conducted at large geographic scales, sustained over a prolonged period of time and control a sufficiently high proportion of rodents [52]. This conclusion is supported by other studies on LASV in Guinea and Sierra Leone [13,30,53].

## Supplementary Material

Supplemental Material
