# Supplementary material for: Households as hotspots of Lassa fever? Assessing the spatial distribution of Lassa virus-infected rodents in rural villages of Guinea
Source: Emerg Microbes Infect. 2020 May 27;9(1):1055–64. doi: 10.1080/22221751.2020.1766381 (PMC7336995; doi:10.1080/22221751.2020.1766381)
Supplement: Supplemental Material [file TEMI_A_1766381_SM2752.zip › SuplementaryTablesandfigures.docx]

**Supplementary tables:**

**S Table 1:** Monte Carlo p-values (based on 1000 simulations) to infer if LASV infected *M. natalensis* (antibody) are significantly clustered in the rodent populations of the villages.

| **Village** | **Date** | **Positives (n)^a^** | **Total (n)^b^** | **N^c^=2** | | **N=4** | **N=6** | **N=8** | **N=10** |
| --- | --- | --- | --- | --- | --- | --- | --- | --- | --- |
| Brissa | 14/12/2013 | 16 | 51 | **<0.01** | **<0.01** | | **<0.01** | **<0.01** | **0.02** |
| Brissa | 26/03/2015 | 8 | 25 | **<0.01** | **0.05** | | **0.01** | NA^d^ | NA |
| Brissa | 9/11/2015 | 18 | 56 | 0.10 | 0.37 | | 0.45 | 0.25 | 0.06 |
| Brissa | 12/11/2016 | 5 | 35 | 1.00 | 1.00 | | NA | NA | NA |
| Dalafilani | 28/11/2013 | 21 | 39 | 0.05 | 0.10 | | 0.30 | 0.34 | 0.56 |
| Dalafilani | 17/04/2014 | 5 | 11 | 0.76 | 0.49 | | NA | NA | NA |
| Dalafilani | 15/01/2016 | 5 | 33 | 0.41 | 0.24 | | NA | NA | NA |
| Dalafilani | 16/01/2017 | 6 | 23 | 0.31 | 0.29 | | 0.26 | NA | NA |
| Damania | 15/11/2013 | 21 | 50 | 0.85 | 0.87 | | 0.52 | 0.08 | **0.01** |
| Damania | 25/03/2014 | 15 | 59 | 0.43 | 0.65 | | 0.63 | 0.93 | 0.99 |
| Damania | 13/11/2015 | 14 | 28 | **0.04** | **0.02** | | 0.15 | **0.05** | **0.04** |
| Damania | 16/11/2016 | 11 | 45 | **0.05** | **<0.01** | | **0.05** | **0.04** | 0.07 |
| Sokourala | 3/04/2013 | 14 | 46 | 0.83 | 0.66 | | 0.66 | 0.64 | 0.71 |
| Sokourala | 31/01/2017 | 17 | 40 | **0.03** | **<0.01** | | **<0.01** | **0.02** | 0.06 |
| Sonkonia | 12/11/2013 | 12 | 46 | **0.05** | **0.02** | | **0.03** | **<0.01** | 0.25 |
| Sonkonia | 7/04/2014 | 7 | 58 | 0.47 | 0.34 | | 0.61 | 0.64 | 0.44 |
| Sonkonia | 19/11/2015 | 8 | 53 | 1.00 | 1.00 | | 1.00 | NA | NA |
| Sonkonia | 24/11/2016 | 16 | 41 | 0.85 | 0.42 | | 0.49 | 0.60 | 0.54 |
| Yarawalia | 20/11/2013 | 8 | 44 | **<0.01** | 0.09 | | 0.15 | NA | NA |
| Yarawalia | 13/04/2014 | 5 | 15 | 0.16 | 0.15 | | NA | NA | NA |
| Yarawalia | 21/03/2015 | 8 | 36 | 0.26 | 0.21 | | 0.26 | NA | NA |
| Yarawalia | 20/11/2016 | 12 | 25 | **0.03** | **<0.01** | | **<0.01** | **<0.01** | **<0.01** |
| All |  | 260 | 859 |  |  | |  |  |  |

^a^ Total number of LASV antibody or PCR positive *M. natalensis*

^b^ Total number of *M. natalensis* captured per trapping session in the villages

^c^ Number of nearest neighbours (*q*)

^d^ Not applicable; bold numbers were considered to be significant.

**S Table 2:** Monte Carlo p-values (based on 1000 simulations) to infer if LASV infected *M. natalensis* (viral RNA) are significantly clustered in the rodent populations of the villages.

| **Village** | **Date** | **Positives (n)^a^** | **Total (n)^b^** | **N^c^=2** | **N=4** | **N=6** | **N=8** | **N=10** |
| --- | --- | --- | --- | --- | --- | --- | --- | --- |
| Brissa | 9/11/2015 | 4 | 56 | 1.00 | 1.00 | NA | NA | NA |
| Brissa | 12/11/2016 | 9 | 35 | 0.14 | 0.07 | **0.02** | **0.03** | NA |
| Dalafilani | 28/11/2013 | 8 | 39 | 0.18 | 0.09 | **0.04** | 0.14 | NA |
| Dalafilani | 17/04/2014 | 3 | 9 | 0.77 | NA | NA | NA | NA |
| Dalafilani | 15/01/2016 | 8 | 33 | 0.08 | **0.03** | 0.08 | 0.07 | NA |
| Dalafilani | 16/01/2017 | 5 | 23 | 0.13 | **<0.01** | NA | NA | NA |
| Damania | 15/11/2013 | 10 | 50 | **0.02** | **0.02** | **0.05** | 0.15 | 0.41 |
| Damania | 25/03/2014 | 11 | 59 | **<0.01** | **0.07** | **<0.01** | **<0.01** | **<0.01** |
| Damania | 13/11/2015 | 3 | 28 | 0.13 | NA | NA | NA | NA |
| Damania | 16/11/2016 | 4 | 45 | **<0.01** | **0.02** | NA | NA | NA |
| Sokourala | 3/04/2013 | 14 | 46 | **<0.01** | **<0.01** | **<0.01** | **<0.01** | **<0.01** |
| Sokourala | 31/01/2017 | 6 | 46 | 0.15 | 0.29 | 0.24 | NA | NA |
| Sonkonia | 7/04/2014 | 4 | 58 | 0.14 | **0.02** | NA | NA | NA |
| Sonkonia | 19/11/2015 | 4 | 53 | **<0.01** | **0.03** | NA | NA | NA |
| Sonkonia | 24/11/2016 | 3 | 41 | 1.00 | 0.30 | 0.24 | 0.13 | 0.06 |
| Yarawalia | 20/11/2013 | 8 | 44 | **0.035** | 0.18 | 0.16 | 0.08 | NA |
| Yarawalia | 20/11/2016 | 3 | 25 | 1.00 | NA | NA | NA | NA |
| All |  | 107 | 609 |  |  |  |  |  |

^a^ Total number of LASV antibody or PCR positive *M. natalensis*

^b^ Total number of *M. natalensis* captured per trapping session in the villages

^c^ Number of nearest neighbours (*q*)

^d^ Not applicable; bold numbers were considered to be significant.

**S table 3:** List with significant local clusters based on the spatial scan statistic for *M. natalensis* that were infected (antibody only) with LASV

| **Village** | **Date** | **P-values** | **Pos/neg^a^** |
| --- | --- | --- | --- |
| Damania | 16/11/2016 | <0.01 | 5/0 |
| Sokourala | 31/01/2017 | <0.01 | 6/1 |
| Sonkonia | 12/11/2013 | 0.05 | 4/0 |
| Yarawalia | 13/04/2014 | 0.01 | 4/2 |
| Yarawalia | 20/11/2016 | <0.01 | 9/1 |

^a^Number of positive versus negative animals that are located within the cluster

**S table 4:** List with significant local clusters based on the spatial scan statistic for *M. natalensis* that were infected (PCR only) with LASV

| **Village** | **Date** | **P-values** | **Pos/neg^a^** |
| --- | --- | --- | --- |
| Damania | 16/11/2016 | <0.01 | 3/1 |
| Sokourala | 3/04/2013 | <0.01 | 8/2 |

^a^Number of positive versus negative animals that are located within the cluster

**Supplementary figures**


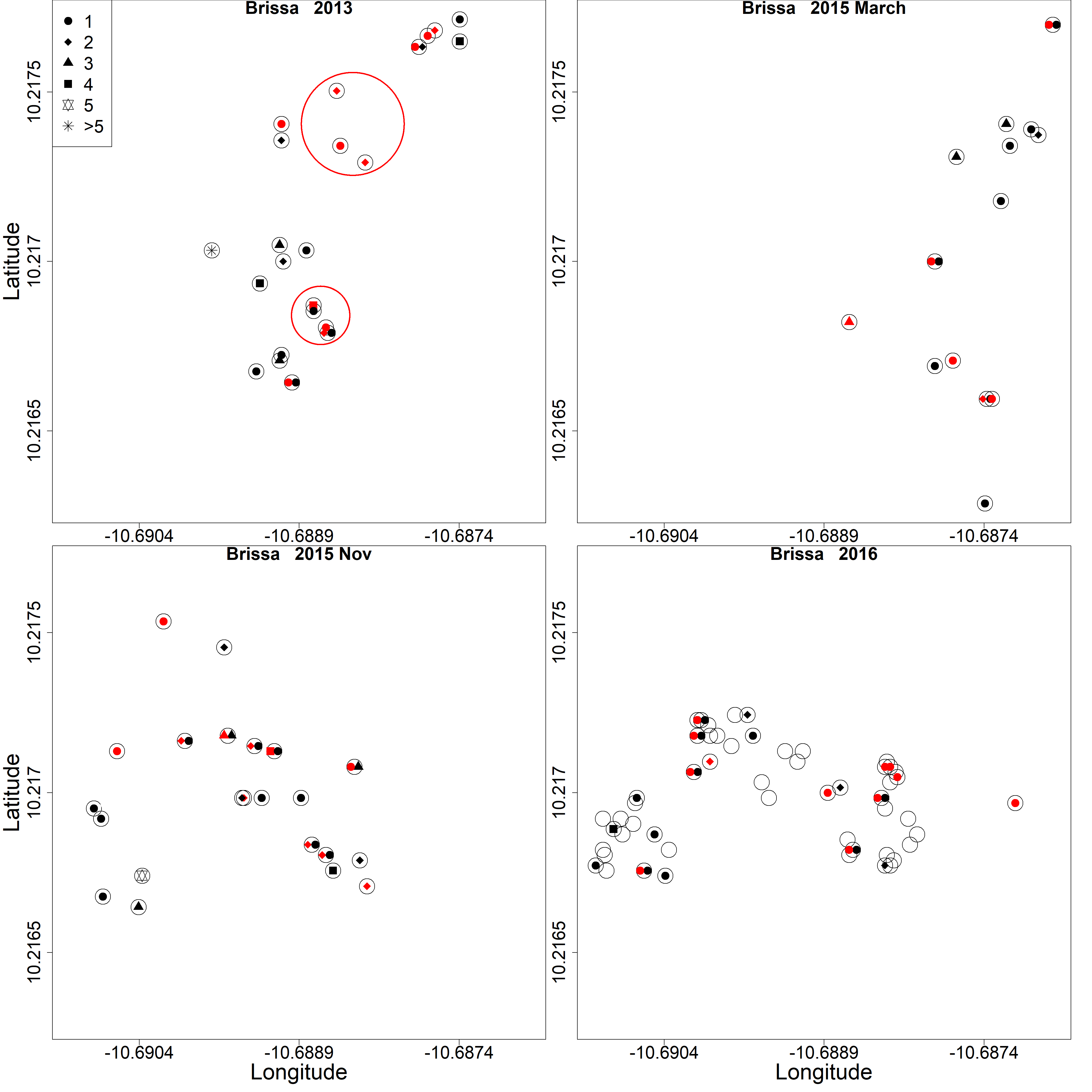


**S Fig 1:** Position of houses (rooms) in the village of **Brissa** where traps were placed. Empty circles represent houses (rooms) where no *M. natalensis* was captured and circles with dots represent houses where *M. natalensis* was captured. Red dots represent LASV-infected individuals (**antibody or PCR-positive**) and black dots uninfected ones. Red circles represent significant clusters of cases based on the spatial statistic scan. Coordinates of houses without rodents were only taken in 2016.


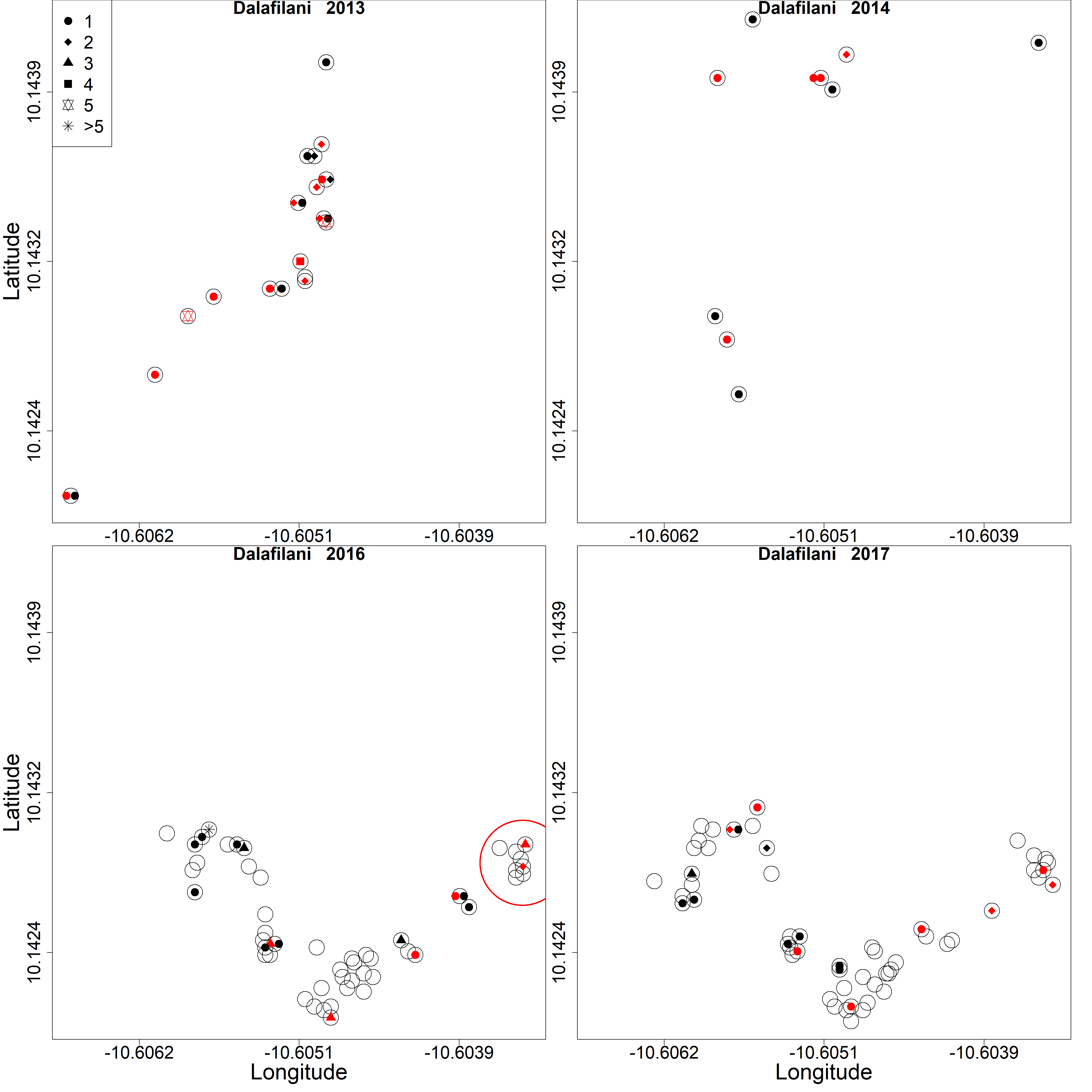


**S Fig 2:** Position of houses (rooms) in the village of **Dalafilani** where traps were placed. Empty circles represent houses (rooms) where no *M. natalensis* was captured and circles with dots represent houses where *M. natalensis* was captured. Red dots represent LASV-infected individuals (**antibody or PCR-positive**) and black dots uninfected ones. Red circle represents a significant cluster of cases based on the spatial statistic scan. Coordinates of houses without rodents were only taken in 2016 and 2017.


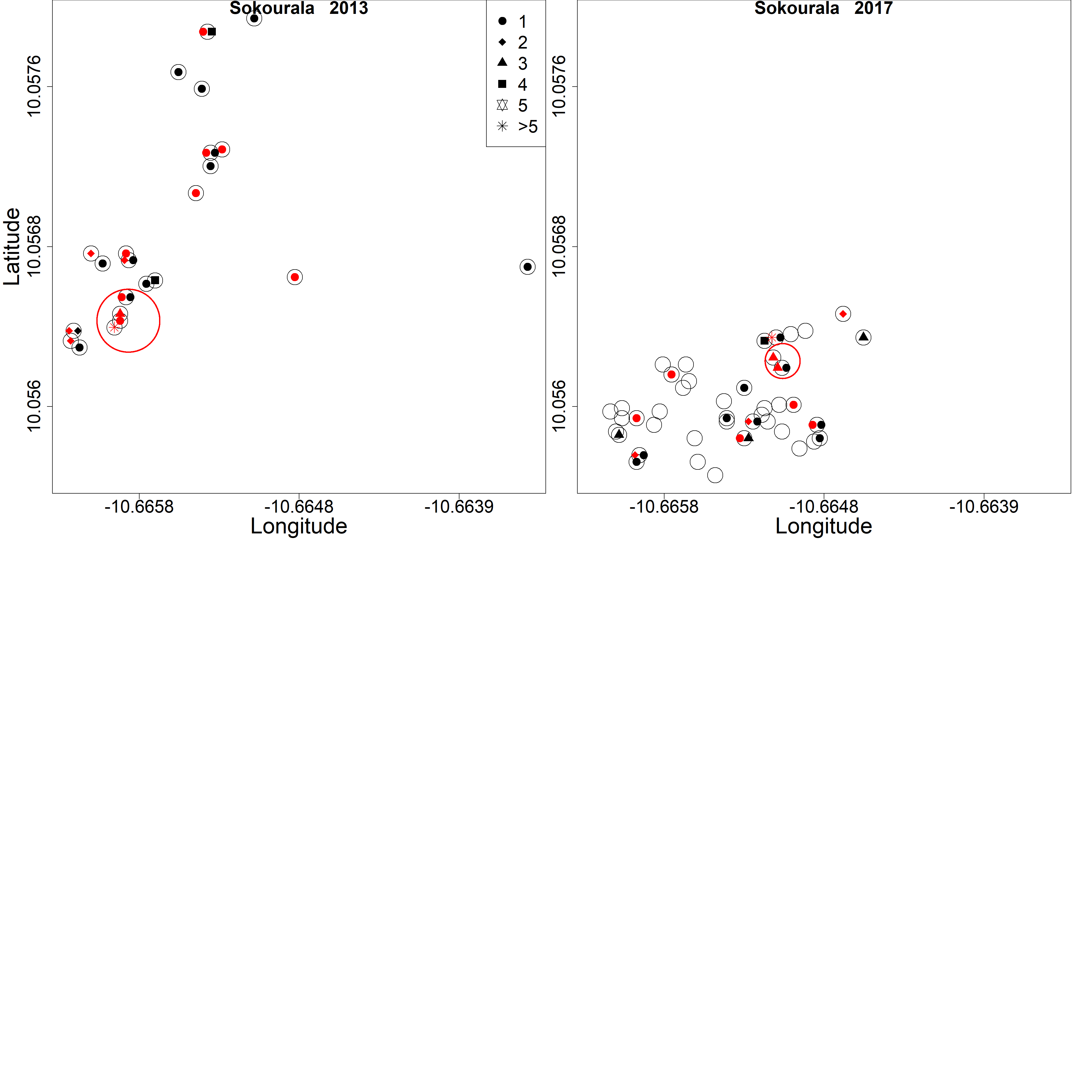


**S Fig 3:** Position of houses (rooms) in the village of **Sokourala** where traps were placed. Empty circles represent houses (rooms) where no *M. natalensis* was captured and circles with dots represent houses where *M. natalensis* was captured. Red dots represent LASV-infected individuals (**antibody or PCR-positive**) and black dots uninfected ones. Red circles represent significant clusters of cases based on the spatial statistic scan. Coordinates of houses without rodents were only taken in 2017.


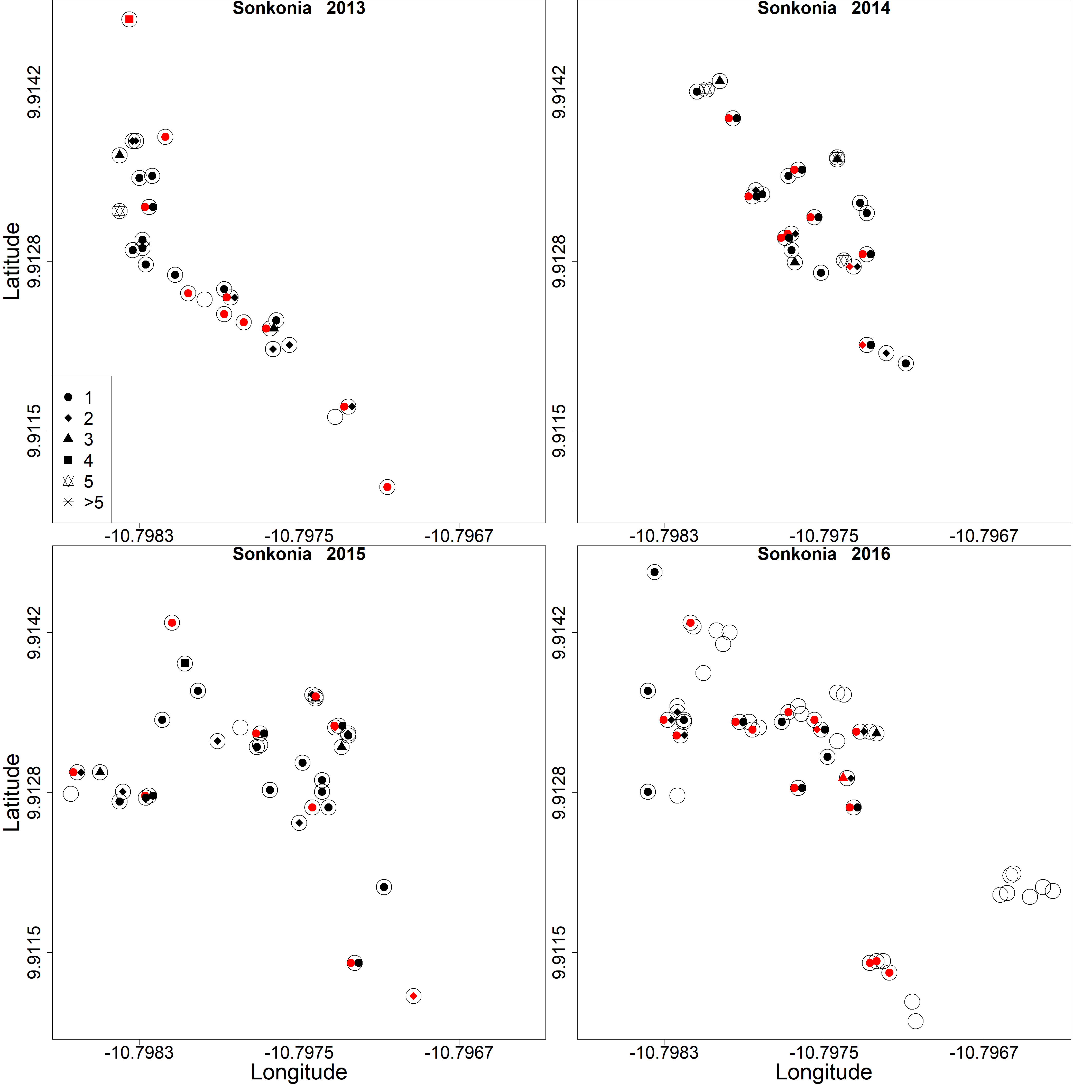


**S Fig 4:** Position of houses (rooms) in the village of **Sonkonia** where traps were placed. Empty circles represent houses (rooms) where no *M. natalensis* was captured and circles with dots represent houses where *M. natalensis* was captured. Red dots represent LASV-infected individuals (**antibody or PCR-positive**) and black dots uninfected ones. Coordinates of houses without rodents were only taken in 2016.


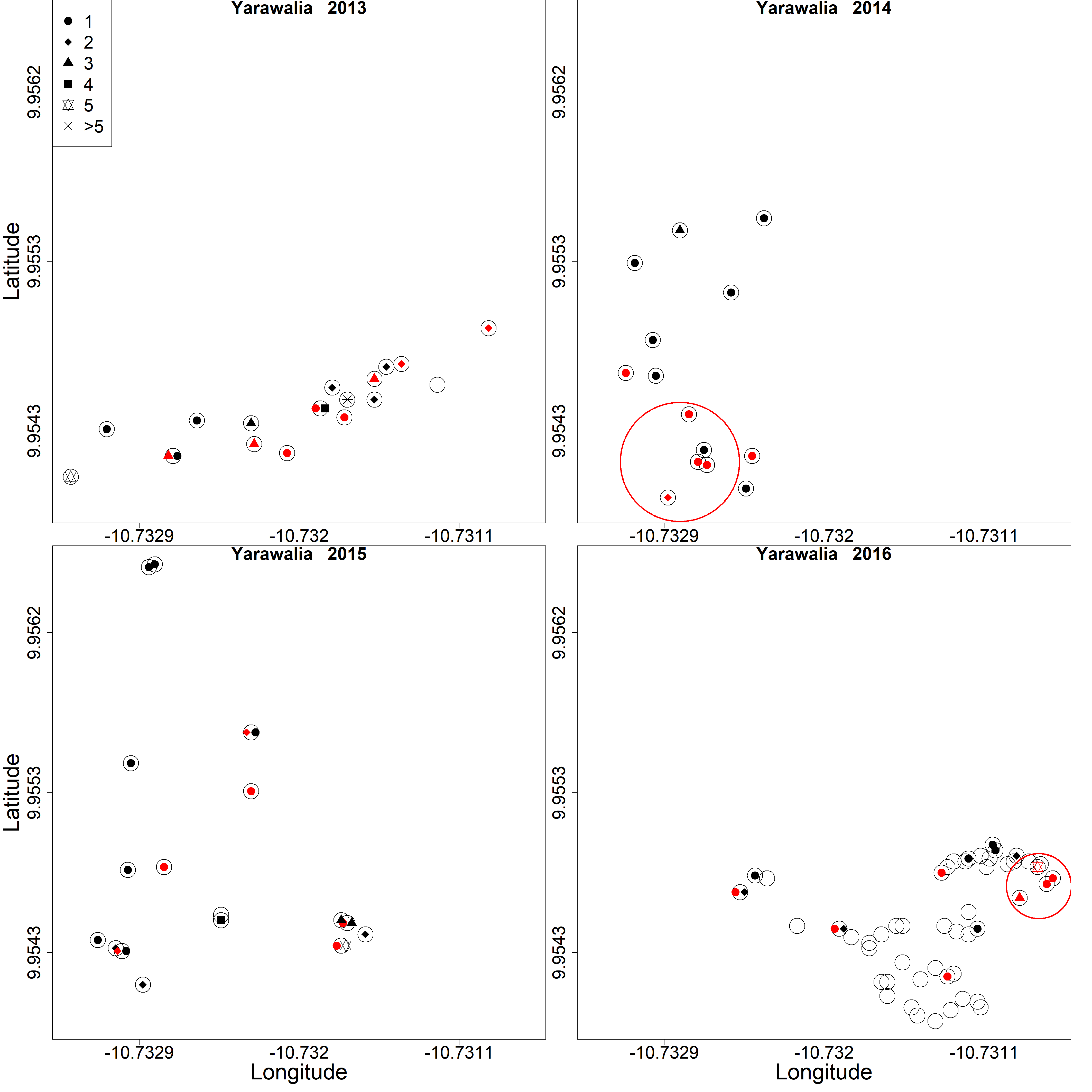


**S Fig 5:** Position of houses (rooms) in the village of **Yarawalia** where traps were placed. Empty circles (rooms) represent houses where no *M. natalensis* was captured and circles with dots represent houses where *M. natalensis* was captured. Red dots represent LASV-infected individuals (**antibody or PCR-positive**) and black dots uninfected ones. Red circles represent significant clusters of cases based on the spatial statistic scan. Coordinates of houses without rodents were only taken in 2016.


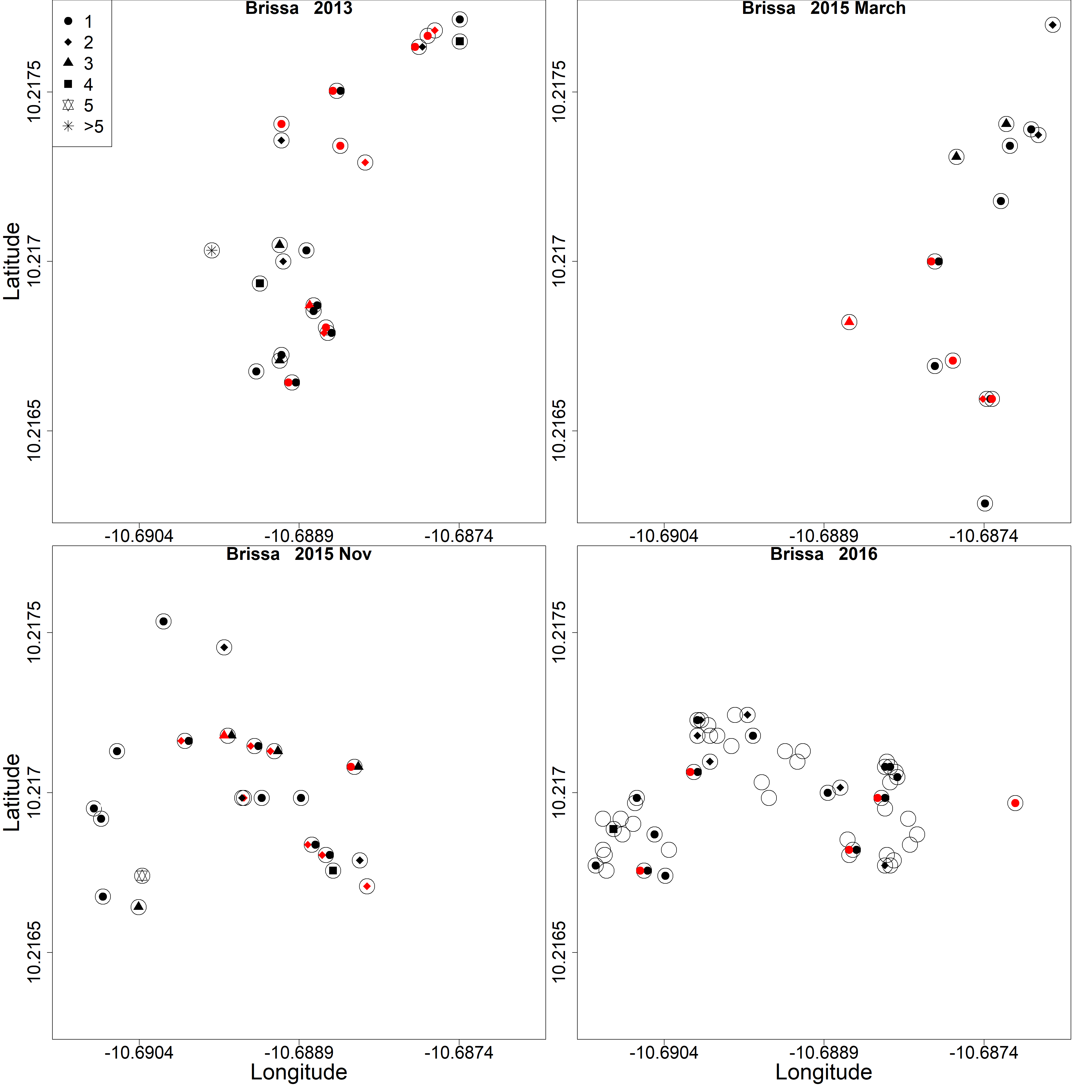


**S Fig 6:** Position of houses (rooms) in the village of **Brissa** where traps were placed. Empty circles represent houses (rooms) where no *M. natalensis* was captured and circles with dots represent houses where *M. natalensis* was captured. Red dots represent LASV-infected individuals (**antibody**) and black dots uninfected ones. Coordinates of houses without rodents were only taken in 2016.


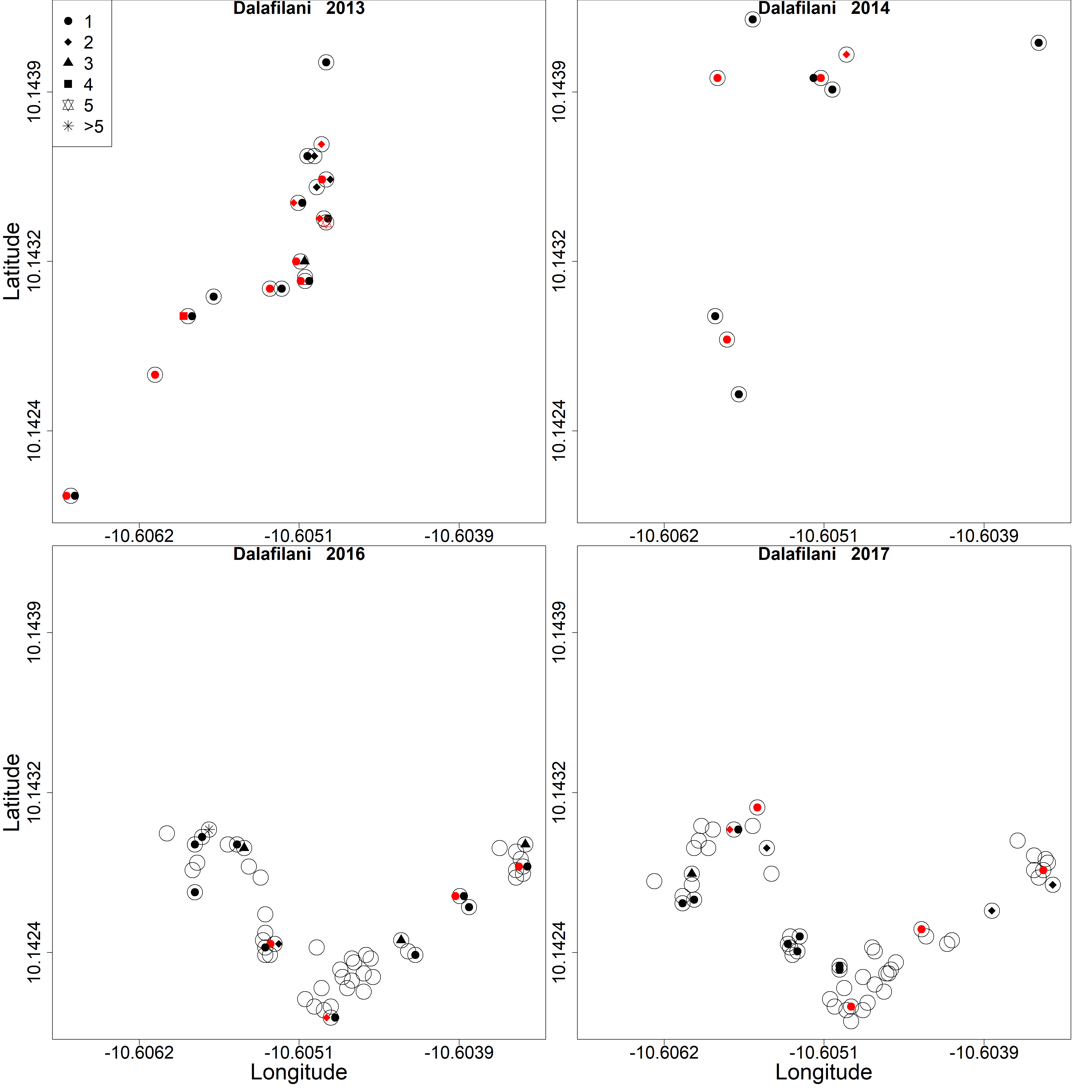


**S Fig 7:** Position of houses (rooms) in the village of **Dalafilani** where traps were placed. Empty circles represent houses (rooms) where no *M. natalensis* was captured and circles with dots represent houses where *M. natalensis* was captured. Red dots represent LASV-infected individuals (**antibody**) and black dots uninfected ones. Coordinates of houses without rodents were only taken in 2016 and 2017.


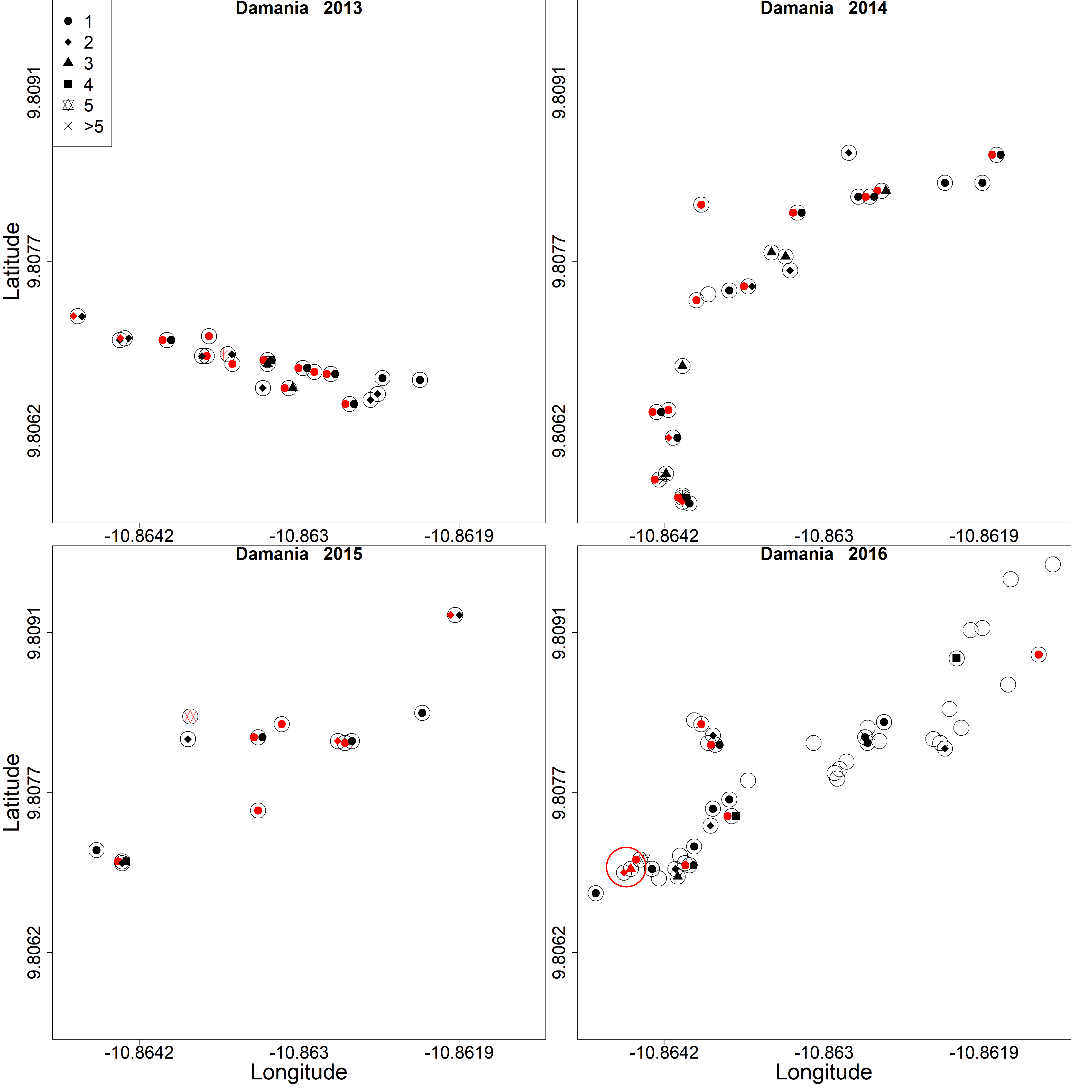


**S Fig 8:** Position of houses (rooms) in the village of **Damania** where traps were placed. Empty circles represent houses (rooms) where no *M. natalensis* was captured and circles with dots represent houses where *M. natalensis* was captured. Red dots represent LASV-infected individuals (**antibody**) and black dots uninfected ones. Red circle represents a significant cluster of cases based on the spatial statistic scan. Coordinates of houses without rodents were only taken in 2016.


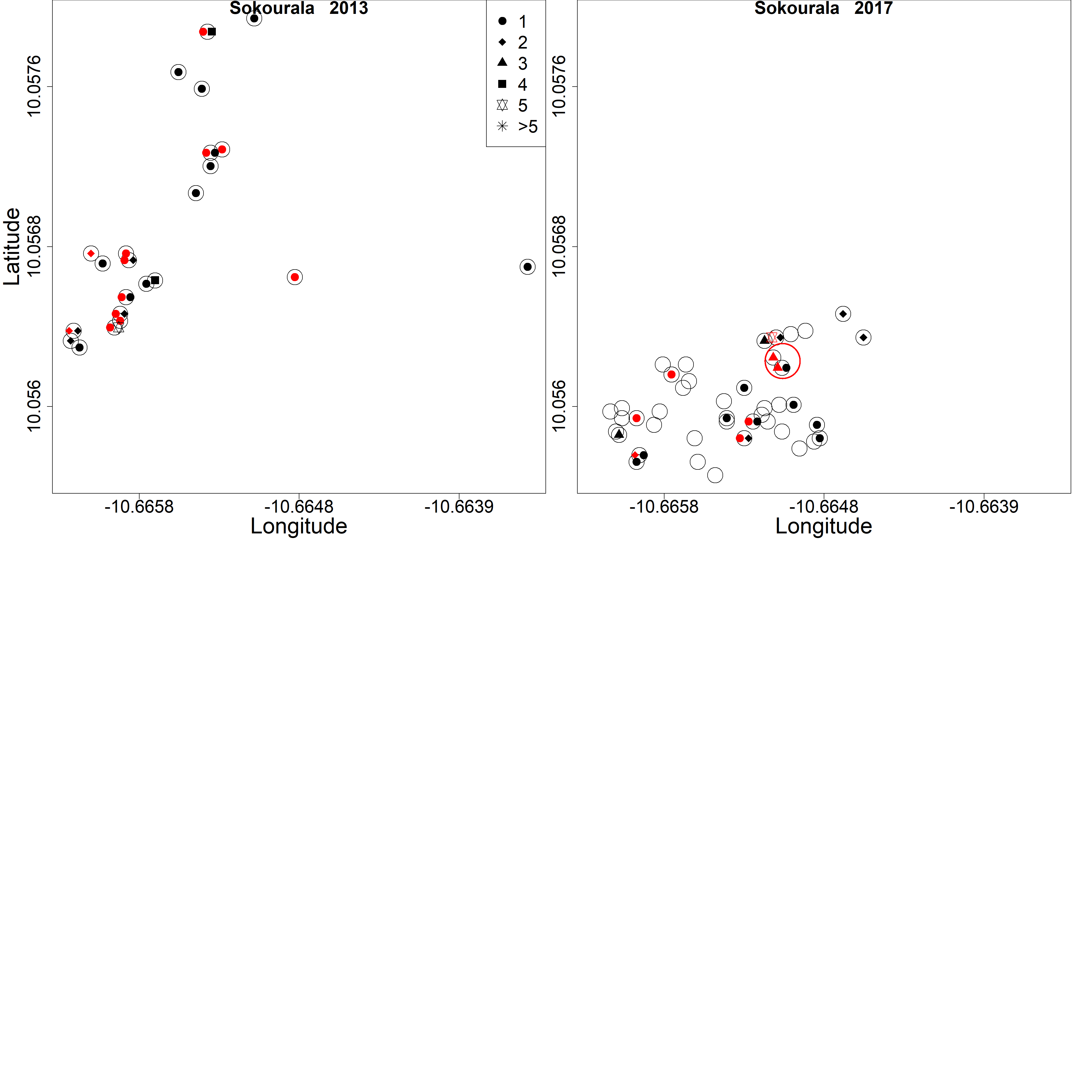


**Fig 9:** Position of houses (rooms) in the village of **Sokourala** where traps were placed. Empty circles represent houses (rooms) where no *M. natalensis* was captured and circles with dots represent houses where *M. natalensis* was captured. Red dots represent LASV-infected individuals (**antibody**) and black dots uninfected ones. Red circle represents a significant clusters of cases based on the spatial statistic scan. Coordinates of houses without rodents were only taken in 2016.


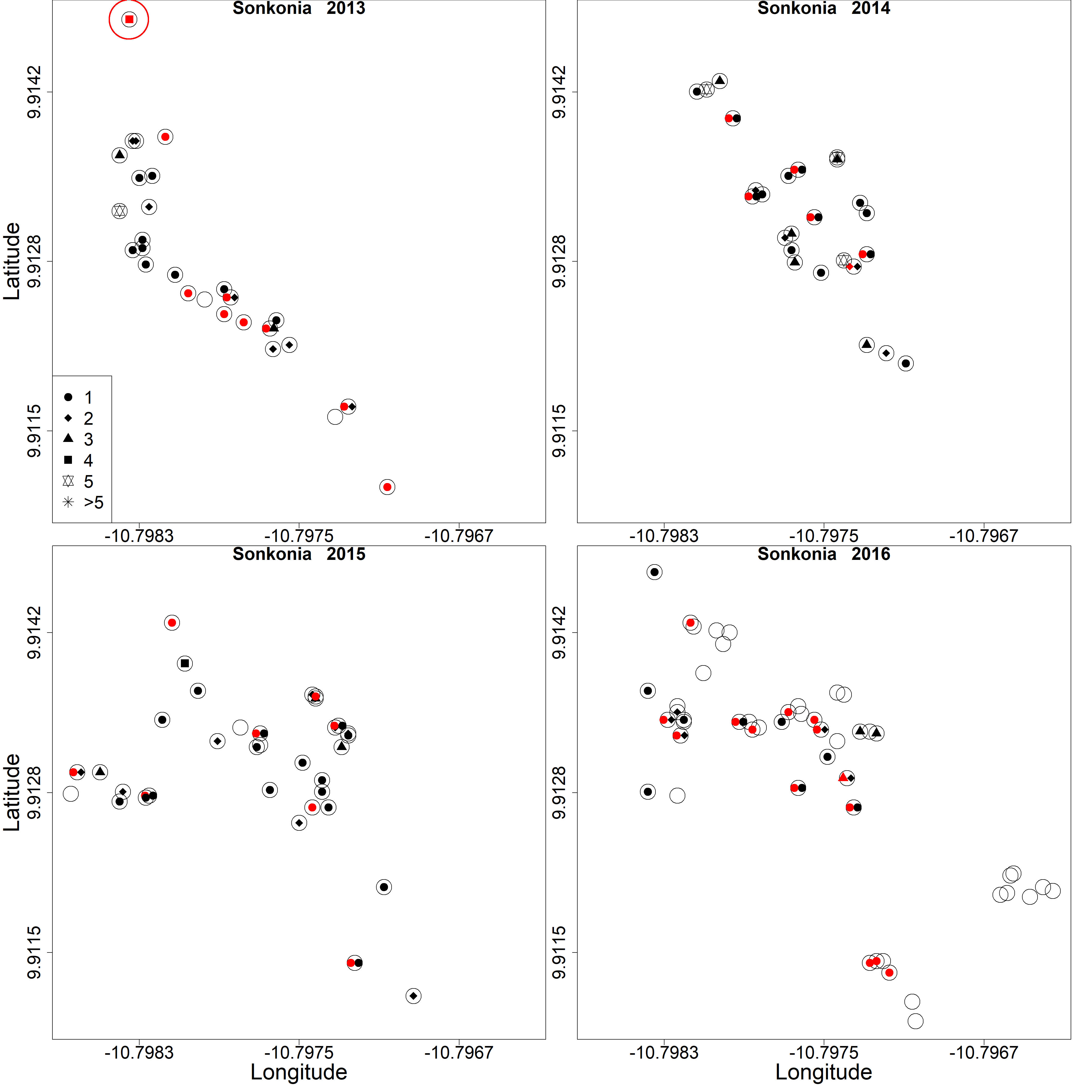


**Fig 10:** Position of houses (rooms) in the village of **Sonkonia** where traps were placed. Empty circles represent houses (rooms) where no *M. natalensis* was captured and circles with dots represent houses where *M. natalensis* was captured. Red dots represent LASV-infected individuals (**antibody**) and black dots uninfected ones. Coordinates of houses without rodents were only taken in 2016.


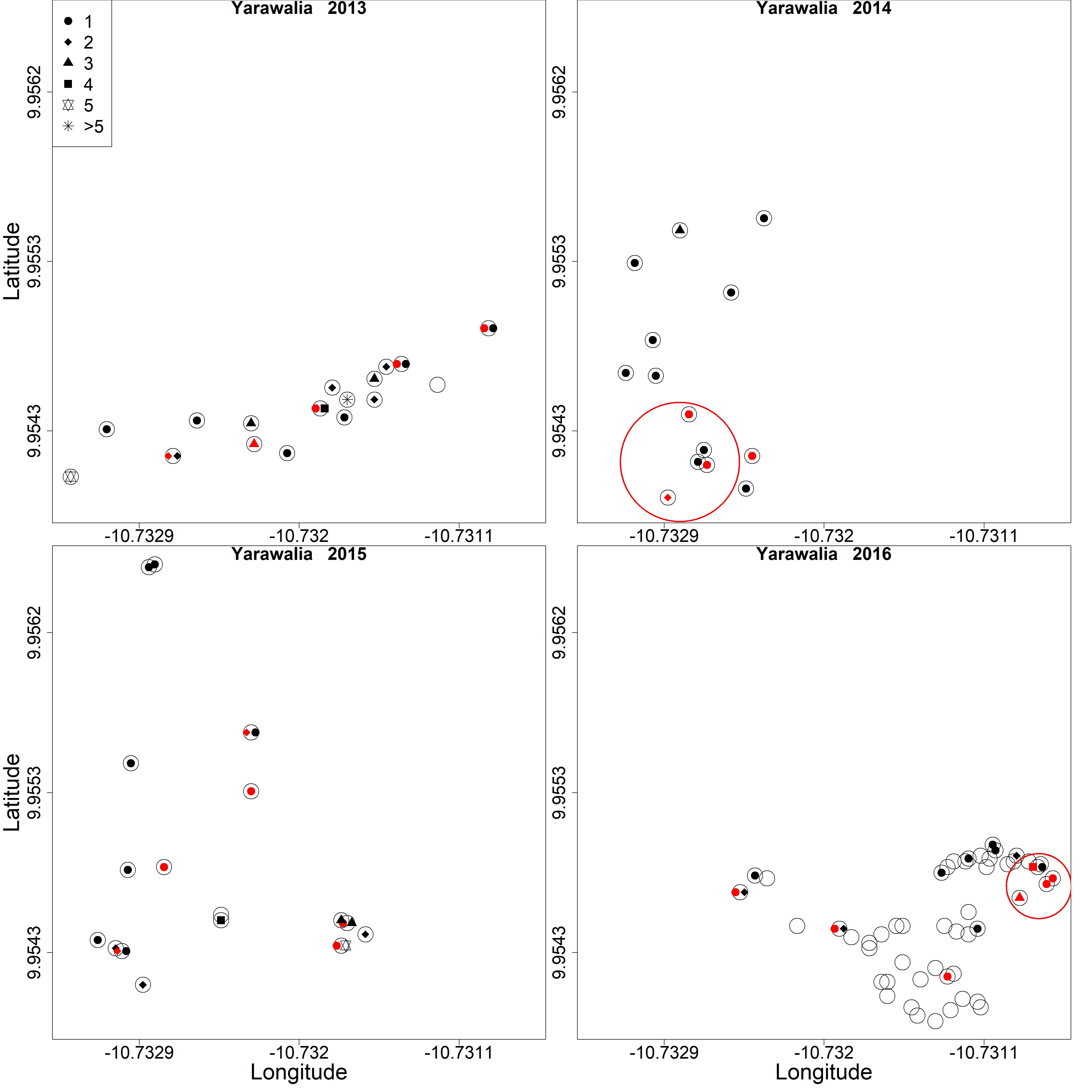


**Fig 11:** Position of houses (rooms) in the village of **Yarawalia** where traps were placed. Empty circles represent houses (rooms) where no *M. natalensis* was captured and circles with dots represent houses where *M. natalensis* was captured. Red dots represent LASV-infected individuals (**antibody**) and black dots uninfected ones. Red circles represent significant clusters of cases based on the spatial statistic scan. Coordinates of houses without rodents were only taken in 2016.


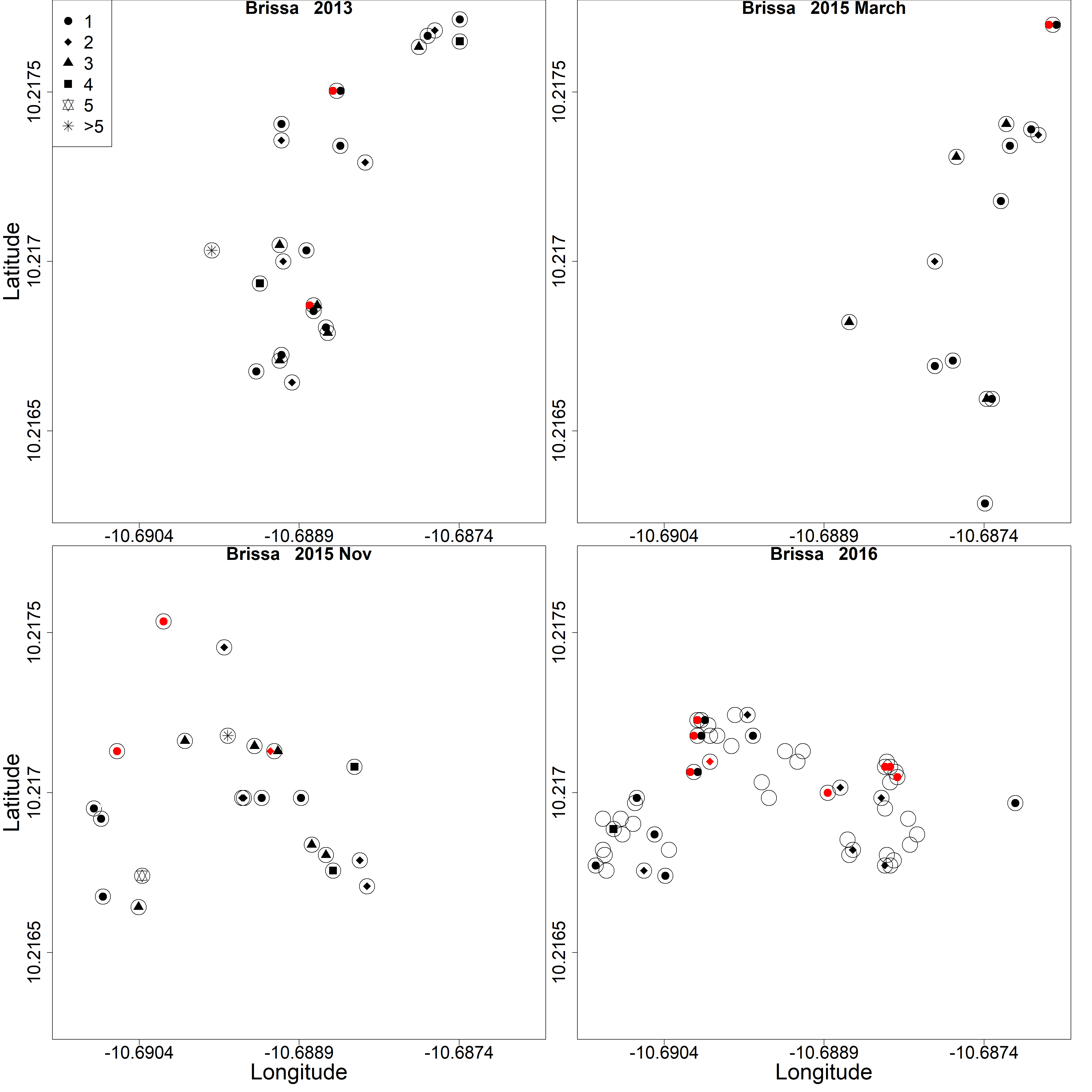


**Fig 12:** Position of houses (rooms) in the village of **Brissa** where traps were placed. Empty circles represent houses (rooms) where no *M. natalensis* was captured and circles with dots represent houses where *M. natalensis* was captured. Red dots represent LASV-infected individuals (**PCR-positive**) and black dots uninfected ones. Coordinates of houses without rodents were only taken in 2016.


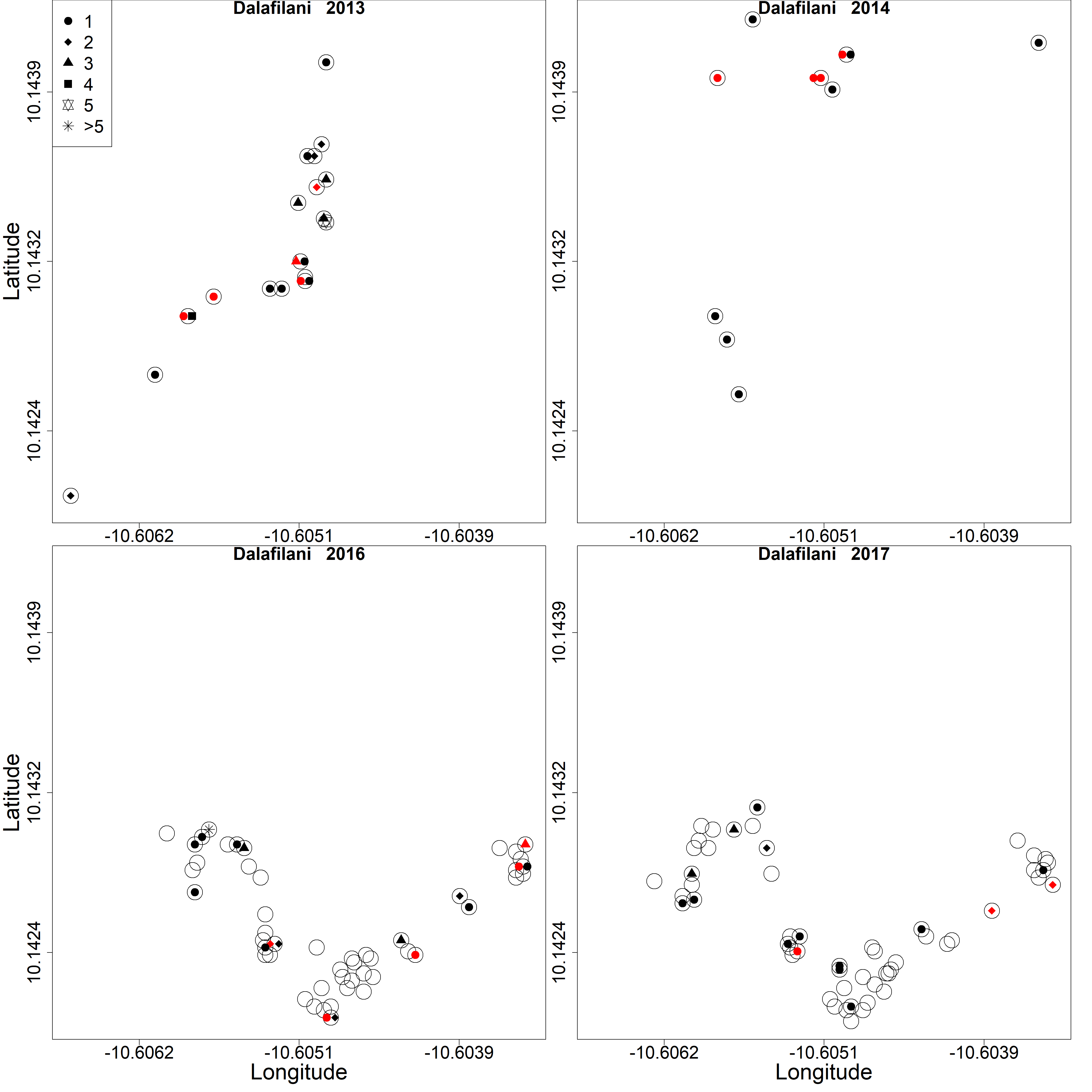


**Fig 13:** Position of houses (rooms) in the village of **Dalafilani** where traps were placed. Empty circles represent houses (rooms) where no *M. natalensis* was captured and circles with dots represent houses where *M. natalensis* was captured. Red dots represent LASV-infected individuals (**PCR-positive**) and black dots uninfected ones. Coordinates of houses without rodents were only taken in 2016 and 2017.


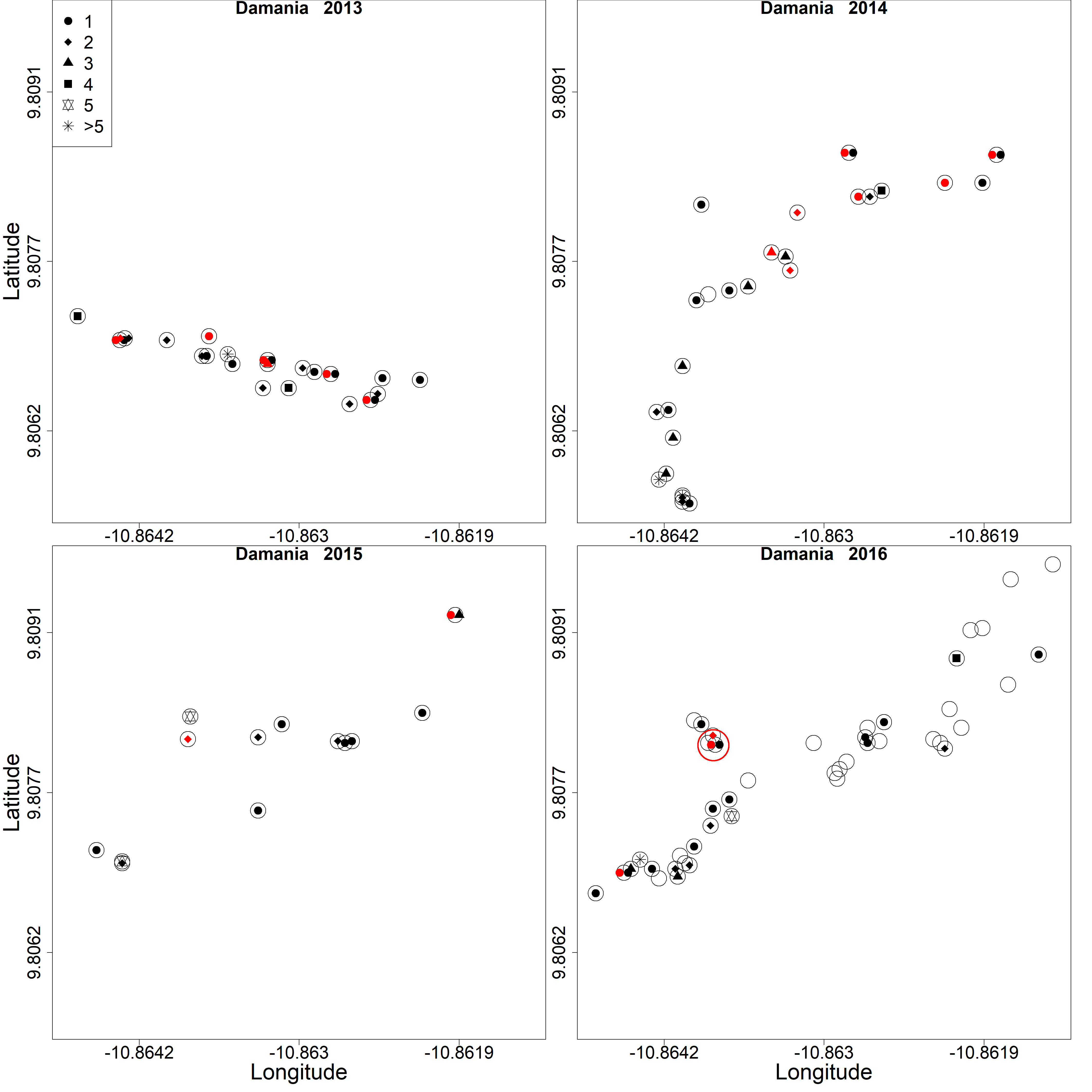


**Fig 14:** Position of houses (rooms) in the village of **Damania** where traps were placed. Empty circles represent houses (rooms) where no *M. natalensis* was captured and circles with dots represent houses where *M. natalensis* was captured. Red dots represent LASV-infected individuals (**PCR-positive**) and black dots uninfected ones. Red circle represent a significant cluster of cases based on the spatial statistic scan. Coordinates of houses without rodents were only taken in 2016.


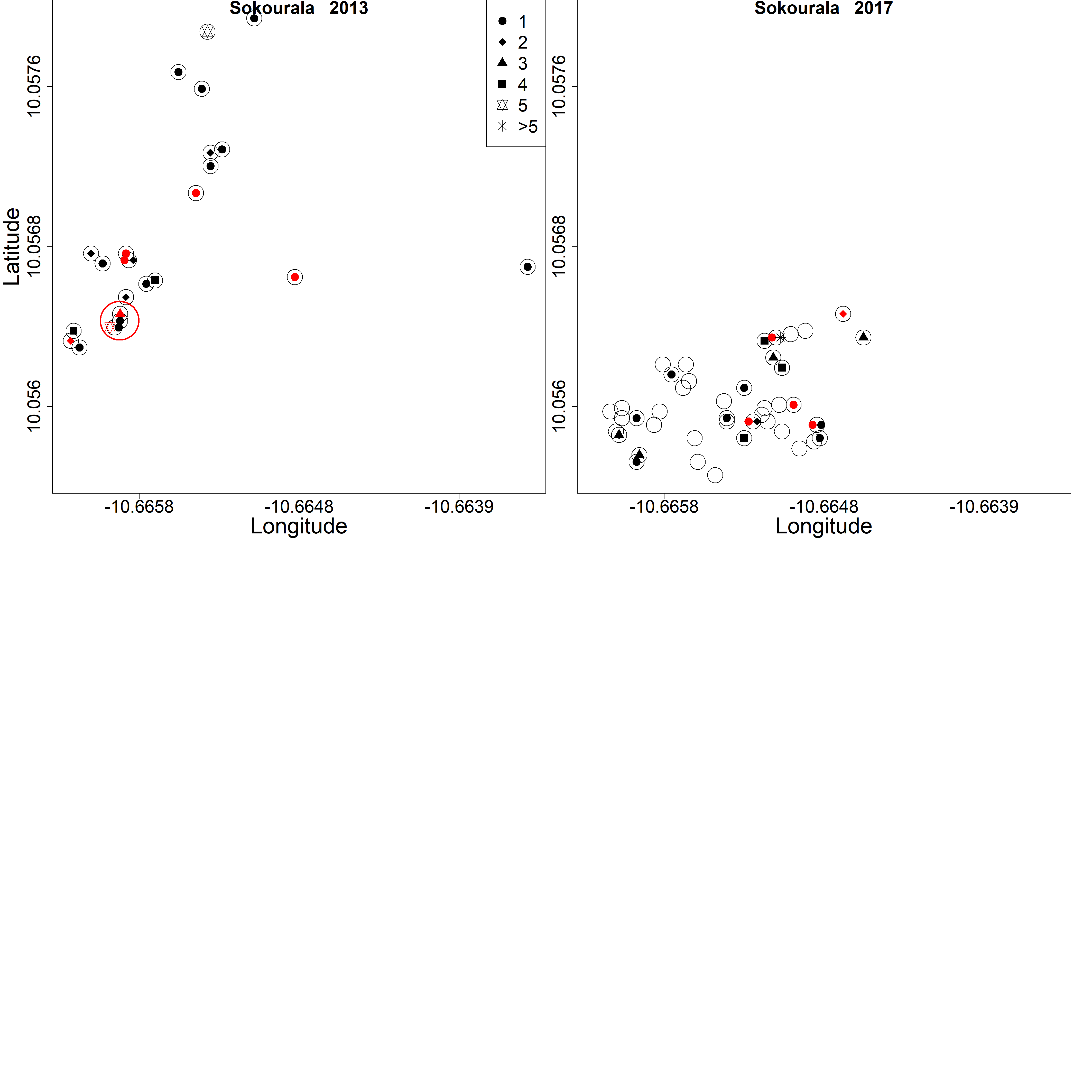


**Fig 15:** Position of houses (rooms)in the village of **Sokourala** where traps were placed. Empty circles represent houses (rooms) where no *M. natalensis* was captured and circles with dots represent houses where *M. natalensis* was captured. Red dots represent LASV-infected individuals (**PCR-positive**) and black dots uninfected ones. Red circle represents a significant cluster of cases based on the spatial statistic scan. Coordinates of houses without rodents were only taken in 2017.


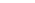


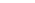


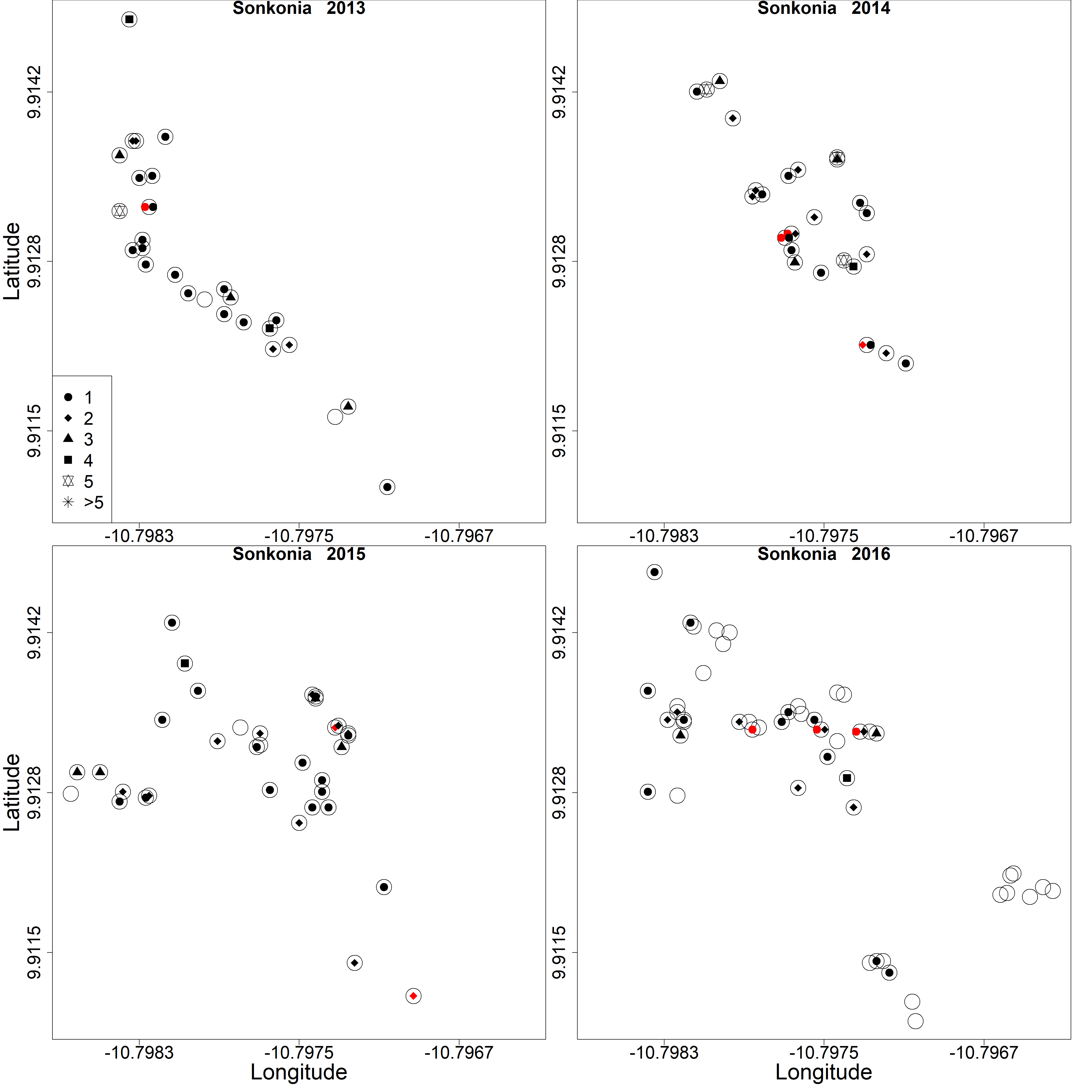


**Fig 16:** Position of houses (rooms) in the village of **Sonkonia** where traps were placed. Empty circles represent houses (rooms) where no *M. natalensis* was captured and circles with dots represent houses where *M. natalensis* was captured. Red dots represent LASV-infected individuals (**PCR-positive**) and black dots uninfected ones. Coordinates of houses without rodents were only taken in 2017.


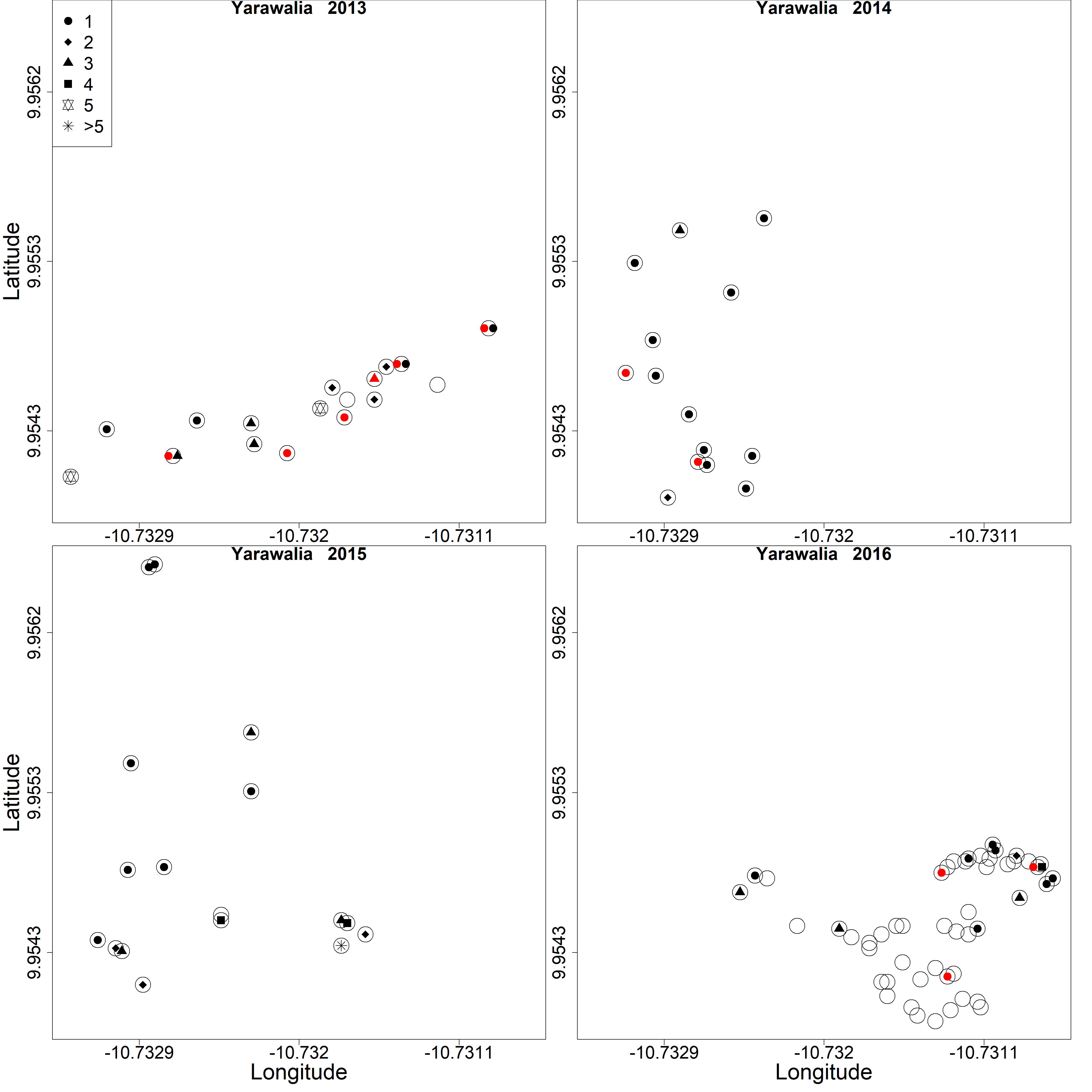


**Fig 17:** Position of houses (rooms) in the village of **Yarawalia** where traps were placed. Empty circles represent houses (rooms) where no *M. natalensis* was captured and circles with dots represent houses where *M. natalensis* was captured. Red dots represent LASV-infected individuals (**PCR-positive**) and black dots uninfected ones. Coordinates of houses without rodents were only taken in 2016.


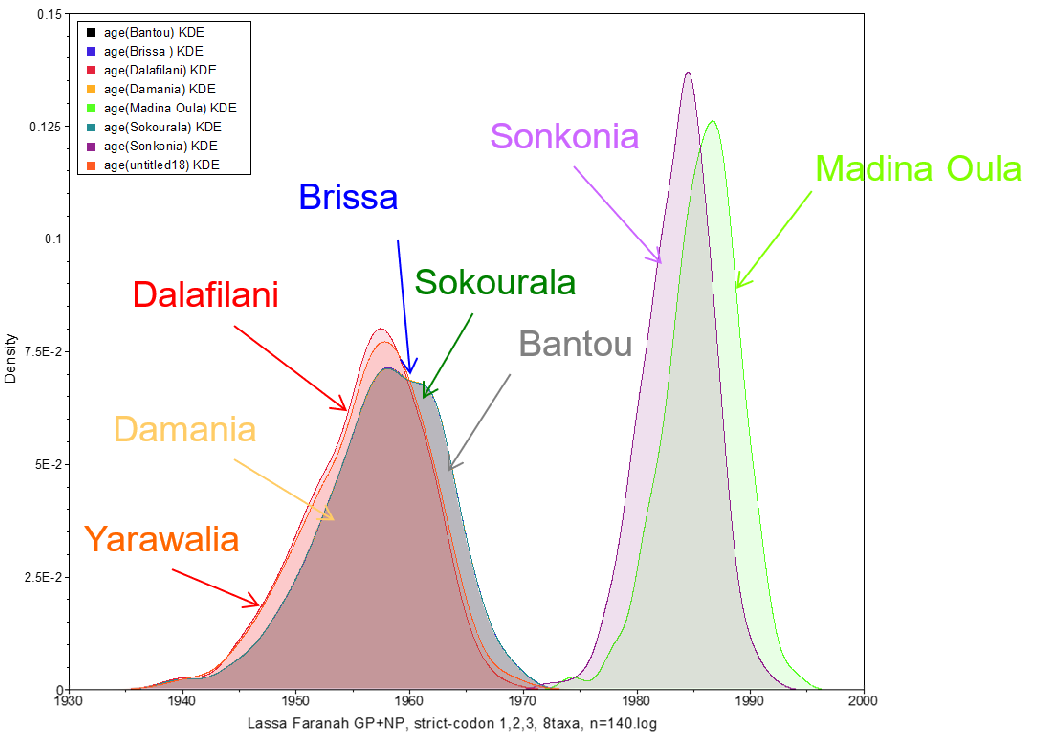


**S Fig 18:** Analysis of the emergence of LASV obtained from *M. natalensis* captured in villages in Guinea. The results suggest a dichotomy between a group of 6 villages where the virus appeared between 1945-1968 [95% confidence intervals of the kernel density estimation (KDE)], and a group of 2 villages where the virus appeared in the 1977-1992. The LASV sequences from the village Madina Oula were obtained from GenBank and used as reference.

**S Fig 19:** Phylogenetic analysis of 140 LASV GP-NP sequences derived from 133 *M. natalensis* (in black), 5 *M. erythroleucus* (in green) and 2 *Homo sapiens* (in pink). The day of capture and village is indicated for each sequence. The tree, based on 888-nucleotide fragment of the GP and 735-nucleotide fragment of the NP gene, was inferred by using the Bayesian Markov Chain Monte Carlo method, with GTR + gamma model, strict clock, constant population size and partitioning into codon positions 1,2,3. Posterior probabilities are coded as follows: 0.80 to < 0.95, pink dots; > 0.95, red dots. The scale axis is coded in years.


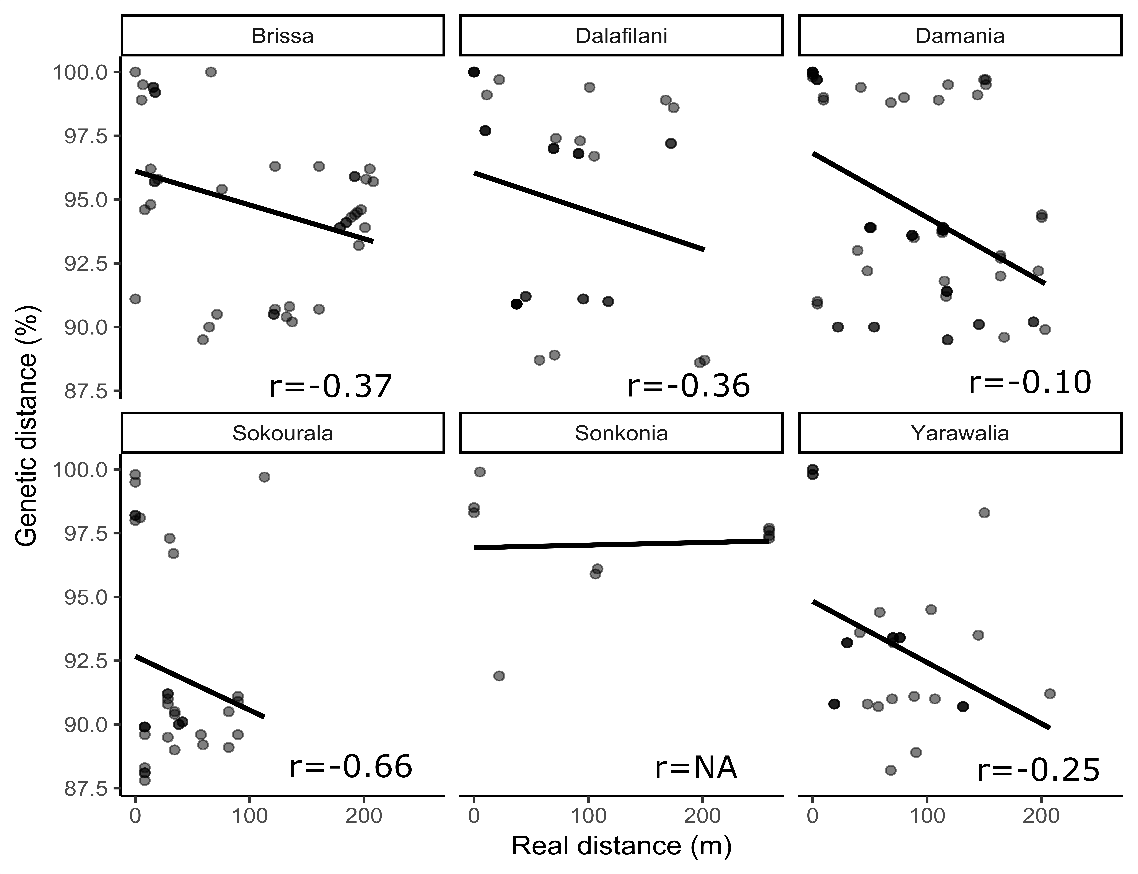


**S Fig 20:** Correlation between the real (Euclidean) and genetic distance (100% = completely similar) for LASV-sequences derived from *M. natalensis* captured during the same trapping session in rural villages in Guinea. Numbers on each panel represent the median correlation coefficient (r) between the real and genetic distance, as explained in the methods section. For Sonkonia, not enough data points were available to calculate a separate mean correlation coefficient.
